# Phosphatase LHPP confers prostate cancer ferroptosis activation by modulating the AKT-SKP2-ACSL4 pathway

**DOI:** 10.1038/s41419-024-07007-8

**Published:** 2024-09-11

**Authors:** Guoqing Xie, Ningyang Li, Keqiang Li, Yating Xu, Yu Zhang, Shun Cao, Budeng Huang, Ruoyang Liu, Peijie Zhou, Yafei Ding, Yinghui Ding, Jinjian Yang, Zhankui Jia, Zhenlin Huang

**Affiliations:** 1 Department of Urology, the First Affiliated Hospital of Zhengzhou University, Zhengzhou, China; 2 Academy of Medical Sciences, Zhengzhou University, Zhengzhou, China; 3 Department of Hepatobiliary and Pancreatic Surgery, the First Affiliated Hospital of Zhengzhou University, Zhengzhou, China

**Keywords:** Prostate cancer, Cell death, Proteomics, Prognostic markers, Screening

## Abstract

LHPP, a novel, recognized tumor suppressor, exerts a critical influence on the regulation of tumor cell proliferation and survival by modulating various signaling pathways with its phosphatase activity. Here, we unveil a robust correlation between reduced LHPP expression and adverse prognosis in prostate cancer. We demonstrate that LHPP interacts with AKT, thereby dampening AKT phosphorylation and subsequently inhibiting ACSL4 phosphorylation at the T624 site. This interaction impedes phosphorylation-dependent ubiquitination, thwarting SKP2 from recognizing and binding to ACSL4 at the K621 site. As a result, ACSL4 is spared from lysosomal degradation, leading to its accumulation and the promotion of lipid peroxidation, and ferroptosis. Moreover, our findings reveal that Panobinostat, a potent histone-deacetylase inhibitor, intricately regulates LHPP expression at multiple levels through the inhibition of HDAC3. This complex modulation enhances the ferroptosis pathway, offering a novel mechanism for curtailing the growth of prostate tumors and highlighting its significant translational potential for clinical application.

## Introduction

Prostate cancer (PCa) holds the second position in global fatality rates among male cancers [1]. The complex mechanisms that trigger the onset of PCa, particularly in high-risk and castration-resistant forms (CRPC), are areas of significant scientific interest. Evidence suggests that the activation of oncogenes and the inactivation of tumor suppressor genes are pivotal in the pathogenesis and progression of PCa [2–5]. In particular, the degradation of LHPP mRNA, which induces AKT phosphorylation, has been linked to the advancement of prostate cancer [6].

Three proteins with pHis phosphatase (PHPs) activity—PHPT1, PGAMS, and LHPP—have been identified [7]. Despite being relatively understudied, histidine phosphatases, particularly LHPP, are gaining recognition for their potential role in cancer progression [8]. AKT, a serine-threonine kinase, plays a crucial role in regulating various biological functions such as cell growth and survival, and is critical to the onset and development of cancer [9]. The absence of LHPP, which leads to amplified AKT activity, is a distinctive feature of the aberrant PI3K/AKT signaling pathway in the genesis and progression of cancer [10–12]. Consequently, LHPP presents as a promising target in cancer therapy.

Ferroptosis, a recently delineated form of programmed cell death, arises from the disruption of lipid peroxidation and the breakdown of antioxidant defenses [13–15]. Acyl-CoA synthetase long-chain family member 4 (ACSL4) is fundamental to ferroptosis, as it orchestrates the synthesis of long-chain fatty acids and influences cellular metabolism [16]. The involvement of ACSL4 in lipid peroxidation and the dismantling of the antioxidant system underpins its role in ferroptosis. It is established that there is a direct correlation between the sensitivity of cells to ferroptosis and the expression levels of ACSL4 [17, 18].

E3 ubiquitin ligases and deubiquitinating enzymes (DUB), exemplified by OTUB1 [19], BAP1 [20], and FBXO10 [21], have been associated with ferroptosis in various human cancers. Ubiquitination, a crucial post-translational modification, is of considerable significance in the development of tumors [22]. The SCF (SKP1-Cullin 1-F-box protein) ubiquitin complex, also referred to as CRL1 (Cullin-RING ligase 1), is able to specifically recognize substrates that have been phosphorylated by kinase [23]. F-Box protein FBXW2 induces S-phase kinase-associated protein 2 (SKP2), a newly identified tumor suppressor in various cancers [24–26]. Importantly, the stability of ACSL4 is regulated by activated AKT [27], although the precise molecular mechanism through which AKT regulates ACSL4 warrants further investigation.

Given the limited extent of prostate cancer drug research conducted in human populations, the results in clinical oncological treatment have been somewhat restricted. Thus, the use of cell line-based drug screening platforms is increasingly gaining attention [28]. The HDAC (Histone Deacetylases) family, known for regulating histone acetylation and gene expression, also modulates the deacetylation and phosphorylation of non-histone proteins. In fact, HDAC inhibitors (HDACi) have been extensively developed for the treatment of various prostate cancers [29]. Panobinostat, a pan-histone deacetylase inhibitor developed by Novartis [30], has been approved for the treatment of multiple myeloma. Current research suggests that Panobinostat has significant potential in the treatment of prostate cancer [31]. Acetylation of the 27th lysine residue of histone H3 can alter the compactness of chromatin, making genes more accessible to transcription factors and RNA polymerases, and thereby increasing gene expression [32]. Thus, H3K27ac is typically associated with gene activation. Research posits that Panobinostat may increase the levels of H3K27ac by inhibiting HDACs, thereby altering gene expression patterns [33].

Our study elucidates a fresh perspective on the anti-prostate cancer mechanism of Panobinostat, affirming the role of LHPP as a tumor suppressor and casting light on the complex interplay of the LHPP/AKT-SKP2-ACSL4 axis in the mechanism of ferroptosis. Specifically, we found that decreased expression of LHPP instigates the activation of the AKT pathway and inhibits ferroptosis, potentially through the phosphorylation of ACSL4 at the T624 site. Once activated, ACSL4 is marked by SKP2 for K63-linked ubiquitination, steering it towards lysosomal degradation, which in turn mitigates lipid peroxidation and ferroptosis. These findings place LHPP in the limelight as an innovative target for prostate cancer therapy, providing a foundation for groundbreaking treatment modalities.

## Results

### Depleted LHPP expression correlates with PCa progression and poor prognosis

**Fig. 1:**
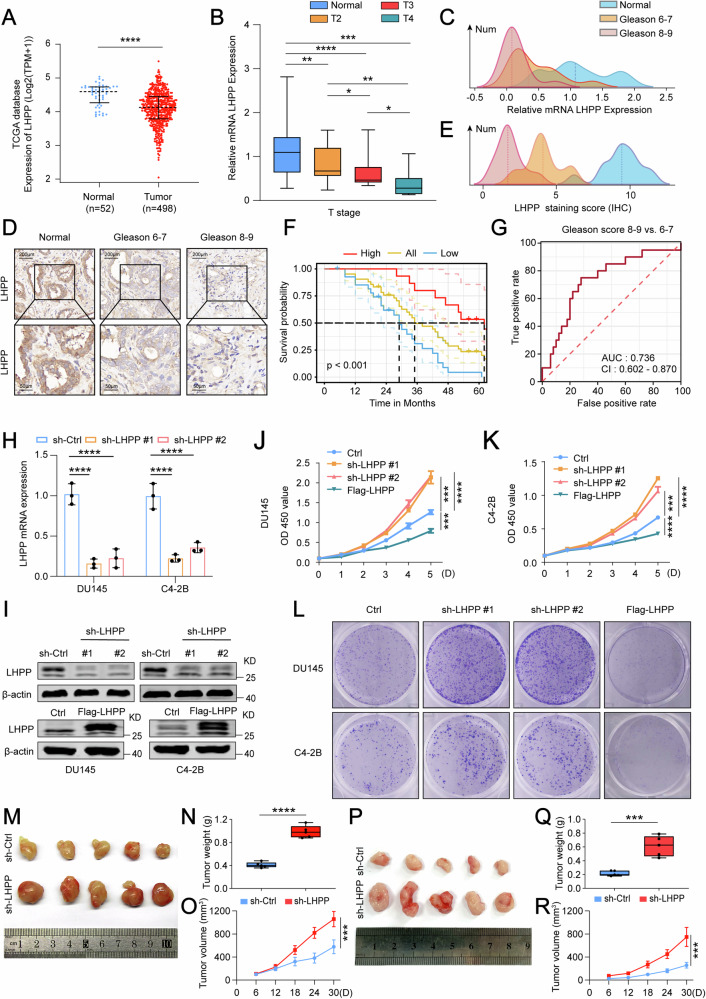
Diminished LHPP expression is linked to the advancement and poorer prognosis in PCa. **A** The significant upregulation of LHPP mRNA levels in normal prostate tissue (*n* = 52) compared to prostate cancer tissue (*n* = 498) as inferred from the TCGA database. **B** The correlation of LHPP expression with the T stage of prostate cancer was assessed through RT-qPCR in 70 prostate cancer patients. **C** The correlation of LHPP expression with the Gleason score of prostate cancer was assessed through RT-qPCR in 70 prostate cancer patients. **D**, **E** IHC staining images depicting a significant reduction in LHPP expression in prostate tissue with a Gleason score of 6-7 and its near absence in tissues with a score of 8–9. **F** Kaplan–Meier survival plots demonstrating a significant correlation between lower LHPP expression and poor prognosis in 70 instances of prostate cancer. **G** ROC curves demonstrate the sensitivity and specificity of LHPP as a marker for PCa prediction. **H**, **I** Effects of LHPP-targeting shRNA and overexpression vector in DU145 and C4-2B cells on expression levels. **J**–**K** CCK-8 assays employed for assessing cell viability in 96-well plates after LHPP knockdown and overexpression in DU145 and C4-2B cells. **L** Colony formation assays in DU145 and C4-2B cells conducted to inspect cell proliferation. **M** Typical images of prostate tumor tissue in nude mice after LHPP knockdown in DU145 cells (*n* = 5). **N** Weight of tumors in each group (*n* = 5). **O** Growth curve of prostate tumor volume in nude mice measured in vivo. **P** Typical images of prostate tumor tissue in nude mice after LHPP knockdown in C4-2B cells (*n* = 5). **Q** Weight of tumors in each group (*n* = 5). **R** Growth curve of prostate tumor volume in nude mice measured in vivo. Statistical significance was determined by unpaired t-test (**A**–**C**, **E**, **H**, **N**, **Q**) or One-way ANOVA (**J**, **K**, **O**, **R**), and data represented as mean ± SD. *p < 0.05; **p < 0.01; ***p < 0.001; ****p < 0.0001, respectively.

Recent explorations have highlighted the crucial role of LHPP in modulating cancer progression [8, 10, 11, 34]. To elucidate the clinical implications of LHPP, we initially analyzed the TCGA database to examine LHPP expression patterns across various human cancers. Notably, LHPP expression was significantly higher in normal prostate tissue than in PCa tissue (Fig. 1A and Fig. S1A). Stratification of 498 PCa specimens based on mean LHPP mRNA expression revealed that patients with low LHPP expression had markedly worse clinical outcomes in terms of T and N stages, and a higher Gleason score than those with high LHPP expression (Fig. S1B). To confirm this observation at the protein level, we performed western blot assays on PCa tissues and adjacent normal tissues, observing a clear reduction in LHPP protein levels in PCa tissues (Fig. S1C, D). To investigate the relationship between LHPP expression and PCa progression, we collected tissue from 70 PCa patients for RT-qPCR and IHC analyses. The results indicated that lower LHPP expression was associated with a higher T stage of PCa compared to normal tissues (Fig. 1B). However, LHPP expression levels did not show significant changes in relation to N and M stages (Fig. S1E, F). Additionally, LHPP may be a crucial determinant in the Gleason score and risk assessment of prostate cancer (Fig. 1C). IHC results supported this, showing significant LHPP presence in normal tissue, marked reduction in tissues with a Gleason score of 6-7, and near absence in tissues with a Gleason score of 8–9 (Fig. 1D, E). Survival analysis of the 70 cases showed a significant link between low LHPP levels and poor prognosis (Fig. 1F, G). We then examined LHPP expression in various prostate cancer cell lines and found that C4-2B and DU145 cells had higher LHPP levels (Fig. S1G, H). These findings led us to select these cell lines for further study of LHPP’s role in PCa progression. We hypothesized that reduced LHPP expression could drive PCa progression. To test this, we designed short hairpin RNA (shRNA) targeting LHPP and assessed the impact of LHPP knockdown on PCa cells (Fig. 1H, I and Fig. S1I). Subsequent EdU proliferation assays confirmed that LHPP silencing increased the number of live EdU-labeled cells in both DU145 (Fig. S2A, B) and C4-2B (Fig. S2C, D) cell lines. Moreover, CCK-8 (Fig. 1J, K) and colony formation assays (Fig. 1L and Fig. S2E) demonstrated that LHPP knockdown significantly enhanced cellular proliferation. We also investigated the effects of LHPP suppression on cell migration and invasion through wound healing and transwell assays, respectively, which indicated that LHPP knockdown notably increased PCa cell migratory (Fig. S2F, G) and invasive capabilities (Fig. S2H, I). In contrast, LHPP overexpression decreased these activities, suggesting that LHPP may play a role in inhibiting cancer progression. To extend our research into an in vivo context, we established subcutaneous xenografts using DU145 (Fig. 1M–O) and C4-2B (Fig. 1P–R) cells with LHPP knockdown in immunodeficient mice. The LHPP-silenced group showed significantly enhanced tumor growth (Fig. 1M–R). IHC analysis revealed increased Ki67 staining in the tumors, indicating higher proliferation due to LHPP knockdown (Fig. S5G, H). Overall, our data support the hypothesis that reduced LHPP expression in PCa tissues is closely associated with disease progression and poorer patient outcomes.

### LHPP promotes ferroptosis in PCa via upregulation of ACSL4 expression

**Fig. 2:**
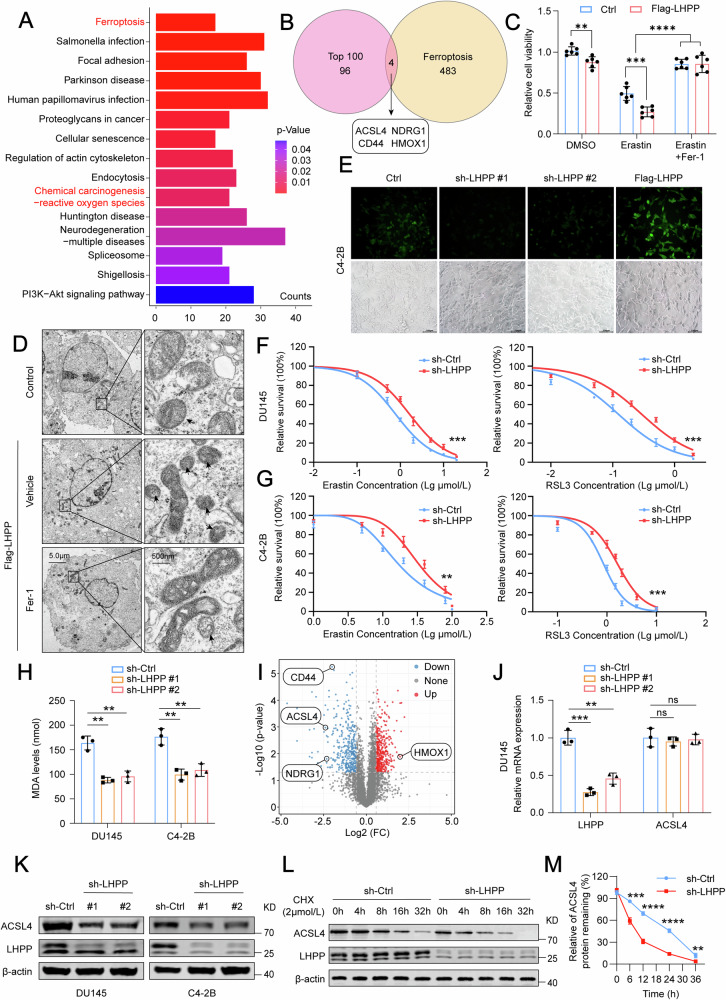
LHPP induces ferroptosis in PCa through the increased expression of ACSL4. **A** The KEGG analysis of Label-Free MS assays in DU145 cells after knockdown of LHPP. **B** Intersection analysis of ferroptosis-related genes and top 100 differentially expressed proteins after LHPP knockdown. **C** Cell viability analysis following Erastin treatment (1 μM) for 24 h in control and LHPP overexpression DU145 cells. **D** The morphological changes of mitochondria were detected by transmission electron microscopy in cells with LHPP overexpression. **E** Representative images showing ROS in the C4-2B cells after LHPP knockdown and overexpression. **F**, **G** IC50 values for Erastin and RSL3 in DU145 and C4-2B cells after LHPP knockdown. **H** Levels of MDA following LHPP knockdown in DU145 and C4-2B cells. **I** The volcano map of Label-Free MS after LHPP knockdown. **J**, **K** RT-qPCR (**J**) and Western blot (**K**) analysis of LHPP and ACSL4 expression after LHPP knockdown. **L** Western blot analysis of half-life of ACSL4 protein after LHPP knockdown followed by treatment with CHX (2 μM) for 0, 4, 8, 16, or 36 h. **M** The protein bands were quantified and normalized to the band intensity at the 0 h time point. Statistical significance was determined by unpaired t-test (**C**, **H**, **J**) or One-way ANOVA (**F**, **G**, **M**) and data represented as mean ± SD. ns not significant; **p < 0.01; ***p < 0.001; ****p < 0.0001, respectively.

To explore potential targets influenced by LHPP, we conducted a proteomic study using Label-Free Mass Spectrometry on DU145 cells after LHPP knockdown and compared them to their corresponding controls. Our subsequent analyses with KEGG and GO revealed that LHPP knockdown significantly activated the ferroptosis and ROS (reactive oxygen species) pathways (Fig. 2A and Fig. S3A), suggesting a possible impact of LHPP alterations on ferroptosis. We then selected the top 100 differentially expressed proteins and 487 ferroptosis-related genes [35] for an intersection analysis, which identified four related proteins: ACSL4, NDRG1, CD44, and HMOX1 (Fig. 2B). Further experiments showed that PCa cells treated with a ferroptosis inducer, Erastin, exhibited pronounced cell death in the control group, but cells with LHPP knockdown were resistant. Conversely, overexpression of LHPP increased the sensitivity to Erastin (Fig. 2C and Fig. S3B). Examination of mitochondrial morphology through electron microscopy revealed significant contraction in cells with increased LHPP, a change that was reversible by the ferroptosis inhibitor Ferrostatin-1 (Fer-1) (Fig. 2D). The knockdown of LHPP resulted in reduced ROS levels, whereas overexpression led to ROS accumulation (Fig. 2E, Fig. S3C). After LHPP knockdown, the IC50 for Erastin and RSL3 increased considerably (Fig. 2F, G), indicating that LHPP expression correlates with the sensitivity of PCa cells to ferroptosis inducers. Moreover, the primary product of ferroptosis, malondialdehyde (MDA), was significantly reduced after LHPP knockdown (Fig. 2H), supporting our hypothesis that LHPP promotes ferroptosis in PCa cells. Furthermore, we conducted IHC staining using markers of ferroptosis, notably MDA and 4-HNE, to validate the activation of ferroptosis in vivo (Fig. S5G, H). Upon further analysis, we found that among the identified proteins, ACSL4 was the most significantly altered after LHPP knockdown (Fig. 2I). RT-qPCR testing of the four genes showed changes at the mRNA level for all except ACSL4, while western blot analysis confirmed protein expression changes (Fig. 2J and Fig. S3D, E). Analysis of PCa tissues from the TCGA database showed that ACSL4 and CD44 were downregulated, whereas NDRG1 and HMOX1 levels remained unchanged (Fig. S3F). Furthermore, the increase in MDA levels following LHPP overexpression was reversible by ACSL4 knockdown but not by the knockdown of HMOX1 or overexpression of NDRG1 and CD44 (Fig. S3G, H). Given the role of ACSL4 in promoting lipid peroxidation, as a key fatty acid synthase in the ferroptosis pathway, particularly when antioxidant defenses are low [17], we created DU145 and C4-2B cell lines with ACSL4 knockdown (Fig. S4A, B), which led to a significant decrease in MDA and ROS levels in PCa cells (Fig. S4C, D). Thus, we propose that ACSL4 is crucial in LHPP-induced ferroptosis. Further, the increase in MDA and ROS caused by LHPP overexpression could be counteracted by ACSL4 knockdown (Fig. S4C, D). These findings suggest that LHPP modulates ferroptosis by regulating ACSL4 expression. Western blot analysis confirmed that ACSL4 protein levels were downregulated after LHPP knockdown (Fig. 2K), while transcription levels remained unchanged (Fig. 2J). Treatment with cycloheximide (CHX, a protein synthesis inhibitor) indicated that the half-life of ACSL4 protein decreased following LHPP knockdown (Fig. 2L, M), while LHPP overexpression stabilized ACSL4 levels and extended its protein half-life (Fig. S4E, F). This implies that LHPP plays a role in enhancing cellular sensitivity to ferroptosis through ACSL4. In conclusion, our findings suggest that LHPP regulates ACSL4 expression, thereby promoting ferroptosis in prostate cancer cells.

### LHPP modulates ACSL4 protein expression by influencing AKT phosphorylation and the ubiquitin-lysosome pathway

**Fig. 3:**
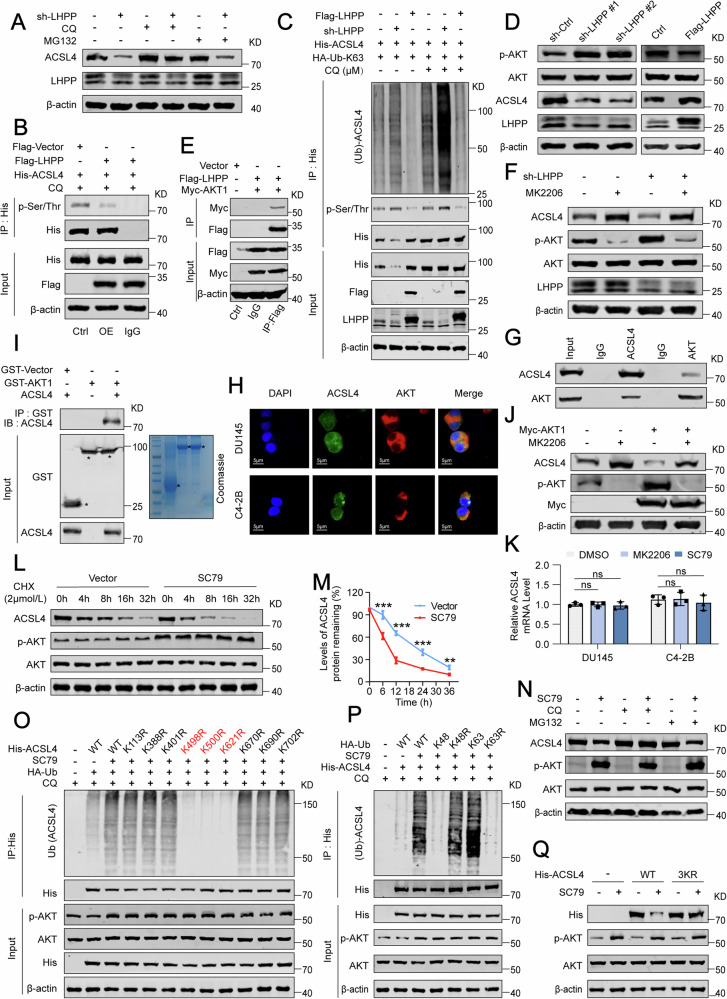
LHPP modulation of AKT phosphorylation and ubiquitin-lysosome degradation affects ACSL4 protein levels. **A** Western blot analysis of the impact of CQ (10 μM) and MG132 (4 μM) for 24 h on ACSL4 reduction mediated by the knockdown of LHPP. **B** The phosphorylation levels of His-tagged ACSL4 in DU145 cells were detected by Co-IP following the overexpression of LHPP and treatment with CQ (10 μM) for 24 h. **C** Western blot analysis of ubiquitination levels of K63 chains in ectopically expressed His-tagged ACSL4 in DU145 cells after the knockdown or overexpression of LHPP and treatment with or without CQ (10 μM) for 24 h. **D** Western blot analysis of activation status of AKT and ACSL4 expression after LHPP knockdown or overexpression in DU145 cells. **E** Western blot analysis of Flag-tagged LHPP’s ability to regulate AKT phosphorylation levels by binding with Myc-tagged AKT1, as identified via co-immunoprecipitation. **F** Western blot analysis of ACSL4 and p-AKT (S473) levels in control and LHPP knockdown cells treated with the AKT inhibitor MK2206 (5 μM) for 24 h. **G** Western blot analysis of endogenous Co-IP revealed an interaction between ACSL4 and AKT. **H** Immunofluorescence analysis of co-localization of ACSL4 and AKT in DU145 and C4-2B cells. **I** Western blot analysis and Coomassie Bright Blue staining of GST pull-down of GST-tagged AKT1 binding with ACSL4 in vitro. **J** Western blot analysis of ACSL4 expression after AKT1 overexpression and treatment with MK2206 (5 μM) for 24 h. **K** RT-qPCR analysis of ACSL4 mRNA after treatment with MK2206 (5 μM) or SC79 (10 μM) for 24 h. **L** Western blot analysis of the half-life of ACSL4 protein after SC79 (10 μM) treatment followed by treatment with CHX (2 μM) for 0, 4, 8, 16, or 36 h. **M** The protein bands were quantified and normalized to the intensity of the band at the 0-h time point. **N** Western blot analysis of ACSL4 level following SC79 treatment (10 μM) and subsequent CQ (10 μM) or MG132 (4 μM) treatment for 24 h. **O** Western blot analysis of ubiquitination of the His-tagged ACSL4 with indicated mutants transfected in HEK-293T cells for 48 h. **P** Western blot analysis of ACSL4 ubiquitination levels after the transfection with different mutant types of ubiquitin for lysis. **Q** Western blot analysis of the expression of wild-type His-tagged ACSL4 and K498/500/621 R mutant after treatment with SC79 (10 μM) for 24 h. Statistical significance was determined by unpaired t-test (**K**) or One-way ANOVA (**M**) and data are represented as mean ± SD. ns not significant; **p < 0.01; ***p < 0.001, respectively.

In the subsequent phase of our research, we aimed to understand how LHPP affects the specific mechanisms of the ACSL4 protein. We treated LHPP-knockdown PCa cells with a proteasome inhibitor (MG132) and a lysosomal inhibitor (CQ). Western blot analysis showed that CQ, but not MG132, prevented the decrease in ACSL4 levels induced by LHPP knockdown (Fig. 3A). To further confirm LHPP’s effect on ACSL4, we exposed LHPP knockdown cells to various lysosomal inhibitors (CQ, NH4Cl, BafA1, and 3-MA), finding that all these inhibitors halted the degradation of ACSL4 following LHPP knockdown (Fig. S4G). Notably, we observed an increase in ACSL4 ubiquitination with K63-linked chains, rather than K48, following co-transfection, suggesting that LHPP modulates ACSL4 stability via the ubiquitin-lysosome pathway (Fig. S4H). Given that LHPP is not an E3 ubiquitin ligase, we further investigated how LHPP affects ACSL4 protein expression. Reviewing the literature, we considered that LHPP might dephosphorylate its substrates [10, 12], leading us to hypothesize that LHPP could alter the phosphorylation status of ACSL4, thereby affecting its protein levels. Initial tests using serine/threonine phosphorylation-specific antibodies for immunoprecipitation and Western blot confirmed LHPP’s role in ACSL4 phosphorylation (Fig. 3B and Fig. S4I). We noted an increase in phosphate-dependent ubiquitin chains (K63-linked) on ACSL4 with reduced LHPP, and conversely, a decrease when LHPP levels were elevated (Fig. 3C). However, a direct interaction between LHPP and ACSL4 was not established (Fig. S4J, K), indicating a potential indirect regulatory mechanism. Considering LHPP’s known ability to inhibit AKT phosphorylation at S473 [6, 10], a modification that may influence ACSL4 expression [27], we speculated that LHPP might regulate ACSL4 through AKT. Our observations confirmed that decreased LHPP correlated with increased phosphorylation of AKT and reduced ACSL4 levels, whereas increased LHPP had the opposite effect (Fig. 3D). Confirming LHPP’s regulation of AKT phosphorylation via immunoprecipitation (Fig. 3E and Fig. S4L), we demonstrated that inhibition of AKT with MK2206 elevated ACSL4 levels, even in the context of diminished LHPP (Fig. 3F). Endogenous Co-IP revealed an interaction between ACSL4 and AKT, supported by immunofluorescence staining showing their co-localization (Fig. 3G, H). In vitro GST pull-down assays confirmed AKT’s direct interaction with ACSL4 (Fig. 3I). Co-IP assays, following exogenous overexpression of ACSL4 and AKT, hinted at a binding between them (Fig. S4M, N). Notably, AKT downregulated ACSL4 protein levels without affecting mRNA expression (Fig. 3J, K). To further examine AKT’s role in modulating ACSL4, we treated cells with an AKT activator, SC79 [36]. This intervention accelerated the degradation of ACSL4 (Fig. 3L, M), yet this effect was abrogated by CQ, but not by MG132 (Fig. 3N). Activation of AKT promoted increased ubiquitination of ACSL4 (Fig. S4O). Inhibition of lysosome, either through CQ treatment or ATG7 gene silencing, prolonged ACSL4 stability (Fig. S5A–D). Co-localization assays using LAMP2 as a lysosomal marker showed significant overlap between ACSL4 and LAMP2 after SC79 treatment (Fig. S5E), suggesting that AKT influences ACSL4 stability through the ubiquitin-lysosome pathway. Upon reviewing the UniProt [37] and GeneCards [38] databases, we pinpointed nine potential ubiquitination sites on ACSL4. By substituting specific lysine residues with arginine, we found that mutations at K498, K500, and K621 significantly reduced ubiquitination in the presence of active AKT (Fig. 3O). Consistently, our results showed that AKT promotes the ubiquitination of ACSL4 primarily through the K63 chains, not the K48 chains (Fig. 3P and Fig. S5F). Moreover, we noted that the degradation of ACSL4 induced by active AKT was prevented when we introduced mutations at the K498, K500, and K621 (Fig. 3Q). IHC analysis of the tumor model showed LHPP knockdown increased AKT phosphorylation and decreased ACSL4 levels (Fig. S5G, H). In summary, our data demonstrate that AKT regulates ACSL4 stability through the ubiquitin-lysosome pathway, with LHPP indirectly modulating this process by influencing AKT phosphorylation.

### E3 ligase SKP2 induces ubiquitination and degradation of ACSL4 protein

**Fig. 4:**
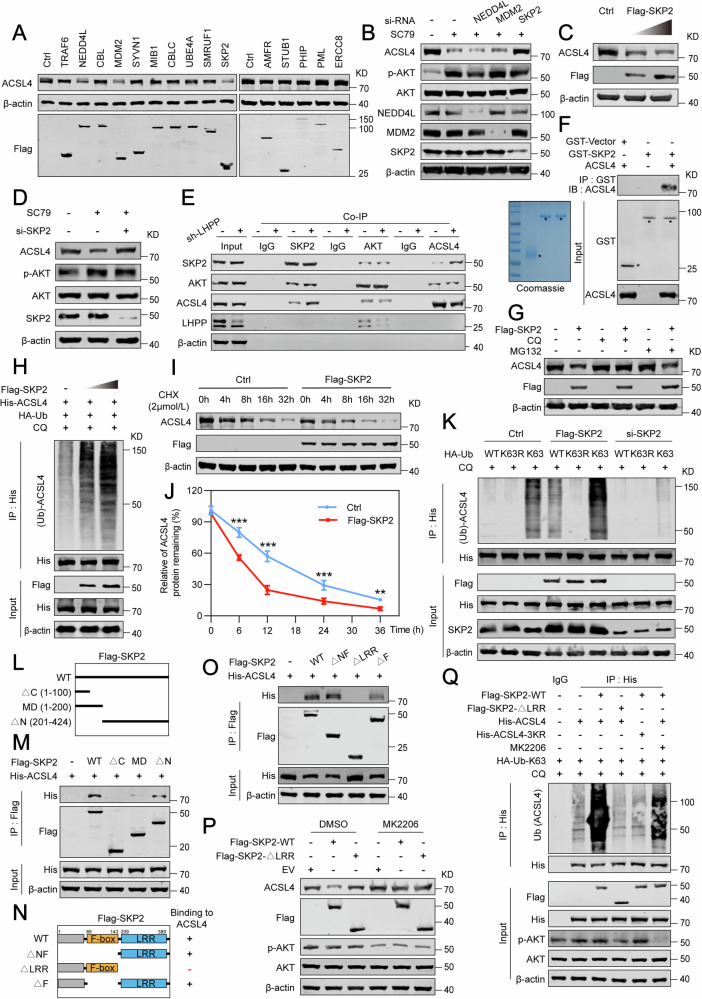
E3 ligase SKP2 induces ubiquitination and degradation of ACSL4 protein. **A** Western blot analysis of ACSL4 expression after ectopic transfection with the indicated E3 ligases in HEK-293T cells. **B** Western blot analysis of ACSL4 expression with SC79 treatment (10 μM) following the knockdown of NEDD4L, MDM2, and SKP2. **C** Western blot analysis of ACSL4 expression in DU145 cells transfected with increasing doses of Flag-tagged SKP2. **D** Western blot analysis of the influence of SKP2 knockdown on SC79-induced (10 μM) ACSL4 degradation. **E** Western blot analysis of endogenous Co-IP revealed an interaction between SKP2, ACSL4, and AKT in LHPP knockdown cells. **F** Western blot analysis and Coomassie Bright Blue staining of GST pull-down of GST-tagged AKT1 binding with ACSL4 in vitro. **G** Western blot analysis of the impact of CQ (10 μM) and MG132 (4 μM) for 24 h on ACSL4 reduction mediated by SKP2. **H** Western blot analysis of the ubiquitination levels of His-tagged ACSL4 shows a dose-dependent increase in DU145 cells overexpressing SKP2 following treatment with CQ (10 μM) for 24 h. **I** Western blot analysis of the half-life of ACSL4 protein after transfection of Flag-SKP2 and treatment of CHX (2 μM) for 0, 4, 8, 16, or 36 h. **J** The protein bands were quantified and normalized to the intensity of the band at the 0 h time point. **K** Western blot analysis of wild-type, K63R, and K63 polyubiquitination levels of His-tagged ACSL4 after SKP2 overexpression or knockdown and treatment with CQ (10 μM) for 24 h. **L** Schematic representation of Flag-tagged wild-type SKP2 and various deletion mutants. **M** Western blot analysis of the IP with a Flag-tagged antibody after the co-transfection of His-tagged ACSL4 and Flag-tagged SKP2 and its various deletion mutants in HEK-293T cells. **N** Schematic representation of Flag-tagged wild-type SKP2 and its various deletion mutants. **O** Western blot analysis of the Co-IP with a Flag-tagged antibody after the co-transfection of His-tagged ACSL4 and Flag-tagged SKP2 and its various deletion mutants in HEK-293T cells. **P** Western blot analysis of ACSL4 expression after the transfection of Flag-tagged wild-type SKP2 and the LRR deletion mutant SKP2 with or without MK2206 treatment (5 μM). **Q** Western blot analysis of the ubiquitination of K63 chains about His-tagged ACSL4 or the K498/500/621R mutant after the transfection of Flag-tagged wild-type SKP2 and the LRR deletion mutant SKP2, with or without MK2206 treatment (5 μM). Statistical significance was determined by One-way ANOVA (**J**) and data are represented as mean ± SD. **p < 0.01; ***p < 0.001, respectively.

We know that AKT isn’t directly responsible for tagging proteins for destruction, we sought to identify which E3 ligases may facilitate AKT-mediated ACSL4 degradation. To this end, we employed the UbiBrowser database [39] to predict the top ten E3 ubiquitin ligases that may interact with both AKT and ACSL4. Subsequent overexpression assays in HEK-293T cells highlighted NEDD4L, MDM2, and SKP2 as principal regulators that reduced ACSL4 protein levels (Fig. 4A). Furthermore, the knockdown of these ligases in the context of AKT stimulation showed that only the knockdown of SKP2 could thwart AKT-induced ACSL4 degradation (Fig. 4B). Subsequent investigations revealed a dose-responsive regulation of ACSL4 by SKP2 (Fig. 4C). In addition, SKP2 knockdown significantly inhibited the SC79-induced degradation of ACSL4 (Fig. 4D and Fig. S5I). Co-immunoprecipitation experiments conducted endogenously unveiled a tripartite interaction between SKP2, AKT, and ACSL4, which was intensified in cells deficient in LHPP (Fig. 4E). In vitro GST pull-down assays indicated that SKP2 directly binds to ACSL4 (Fig. 4F). Consistently, there was an interaction between ectopically introduced AKT with SKP2, and between SKP2 and ACSL4 (Fig. S5J, K). The disruption of this interaction by CQ, as opposed to MG132 (Fig. 4G), along with the increased co-localization of ACSL4 with the lysosomal marker LAMP2 following SKP2 overexpression (Fig. S5L), emphatically suggests the lysosomal pathway’s involvement in ACSL4 stability regulation by SKP2. Concurrently, assessing the ubiquitination levels of ACSL4 in prostate cancer cells, we ascertained that SKP2’s overexpression markedly amplified ACSL4 ubiquitination in a dose-correlated manner (Fig. 4H). Additionally, an enhanced presence of SKP2 significantly shortened the half-life of ACSL4 protein (Fig. 4I, J). Further research revealed that SKP2 overexpression increased both wild-type and K63 chains ubiquitination of ACSL4 but had no effect on the ubiquitination of K63R mutants, whereas SKP2 silencing reduced K63 chains ubiquitination of ACSL4 (Fig. 4K). To understand how SKP2 interacts with ACSL4, we constructed three truncated SKP2 mutants: a C-terminal deletion (∆C), a middle domain variant (MD), and an N-terminal deletion (∆N) (Fig. 4L). Our analyses indicated that the ∆C mutant failed to bind ACSL4 (Fig. 4M). Further domain dissection led to the creation of three additional SKP2 mutants: (∆NF), a leucine-rich repeat (LRR) domain deletion (∆LRR), and an F-box motif deletion (∆F) (Fig. 4N). Our findings demonstrated that deletion of the LRR domain (∆LRR) abrogated SKP2’s binding to ACSL4 (Fig. 4O), underscoring the indispensability of the SKP2-LRR (but not the F-box motif necessary for SCF complex formation) domain for this interaction. This conclusion was supported by the fact that only the wild-type SKP2, not the ∆LRR mutant, effectively reduced ACSL4 protein levels and increased K63-linked ubiquitination, whereas mutations at the key residues K498, K500, and K621 negated these effects (Fig. 4P, Q). In summary, our research emphasizes that AKT binds to ACSL4 and, in a manner dependent on SKP2, increases the K63-linked ubiquitination of ACSL4, with the SKP2-LRR domain necessary for the binding to ACSL4, resulting in the targeting of ACSL4 for degradation via the lysosomal pathway.

### AKT-induced phosphorylation at ACSL4’s T624 site leads to SKP2-mediated ubiquitin degradation of ACSL4

**Fig. 5:**
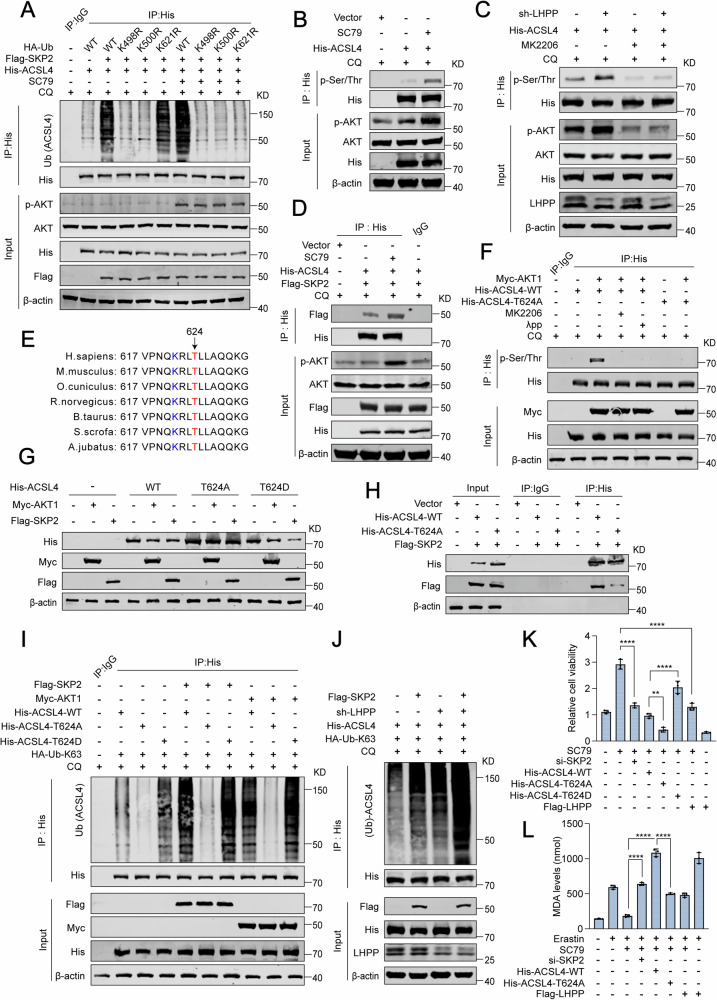
AKT-induced phosphorylation at ACSL4’s T624 site leads to SKP2-mediated ubiquitin degradation of ACSL4. **A** Western blot analysis of the ubiquitination of His-tagged ACSL4 with the indicated mutants transfected into HEK-293T cells for 48 h, with or without SC79 treatment (10 μM). **B** The phosphorylation status of His-tagged ACSL4 was detected by Co-IP after treatment with SC79 (10 μM) for 24 h. **C** The phosphorylation levels of His-tagged ACSL4 in DU145 cells were detected by Co-IP following LHPP knockdown and treatment with CQ (10 μM) for 24 h. **D** The interaction between Flag-tagged SKP2 and His-tagged ACSL4 was detected by Co-IP under SC79 (10 μM) treatment for 24 h and CQ (10 μM) treatment for 24 h. **E** Schematic representation of the conservation of ACSL4 T624 among different species. **F** The phosphorylation levels of His-tagged ACSL4 under the indicated conditions. **G** Western blot analysis of His-tagged ACSL4 expression after transfection with the indicated ACSL4 mutant. **H** Western blot analysis of the interaction between Flag-tagged SKP2 and His-tagged ACSL4 detected by Co-IP. **I** Western blot analysis of the ubiquitination of His-tagged ACSL4 under the indicated conditions. **J** Promotion of SKP2-mediated multi-ubiquitin degradation of ACSL4 at the K63 site following LHPP knockdown. **K** CCK-8 assay to test cell viability in DU145 cells transfected with the indicated vectors. **L** MDA assay to test lipid peroxidation in DU145 cells transfected with the indicated vectors. Statistical significance was determined by unpaired t-test (**K**, **L**) and data are represented as mean ± SD. ***p < 0.001; ****p < 0.0001, respectively.

Interestingly, the suppression of AKT activity mitigates the ubiquitination and subsequent degradation of ACSL4 instigated by SKP2 (Fig. 4P, Q and Fig. S5M). Further investigation revealed that in the absence of AKT activation, only the K498R and K500R mutations countered the ubiquitination of ACSL4 by SKP2. Contrarily, when AKT was active, mutations at K498R, K500R, and K621R all diminished ACSL4 ubiquitination (Fig. 5A). Considering AKT’s role as a serine/threonine kinase, we hypothesized that AKT activation induces a phosphorylated state of ACSL4, particularly enhancing activity at the K621 site and thus facilitating its recognition by SKP2, which in turn promotes ubiquitin-mediated degradation of ACSL4. To test this, we examined whether AKT phosphorylates ACSL4 and observed a significant alteration in ACSL4’s phosphorylation profile post-treatment with SC79 and MK2206, compared to the control (Fig. 5B and Fig. S6A). Collectively, these findings collectively support the notion that LHPP modulates ACSL4 in a manner responsive to AKT (Fig. 5C). Delving into the interactions among AKT, ACSL4, and SKP2, we found that SC79 treatment bolsters the SKP2-ACSL4 interaction, implying that AKT activation may fortify this nexus (Fig. 5D). Additionally, SC79 treatment appears to intensify SKP2-mediated ubiquitination and degradation of ACSL4 (Fig. S6B). These observations led us to deduce that SKP2-mediated degradation of ACSL4 at the K621 site is contingent upon AKT-induced phosphorylation of ACSL4. Using the Scansite [40] database, we identified several potential AKT phosphorylation sites on ACSL4, including S57, S61, S447, and T624 (Data not shown). Given that AKT can trigger SKP2’s recognition of ACSL4 at the K621 site for ubiquitination, we posited that AKT-mediated activation at T624 might influence the activity at the K621 site, rendering it more susceptible to SKP2. Sequence analysis confirmed the high conservation of ACSL4’s T624 across various species (Fig. 5E). Subsequently, we examined AKT’s role in promoting ACSL4 phosphorylation, noting that this effect was nullified when the threonine at position T624 was replaced with alanine (T624A, a phosphorylation-deficient mutant) or when treated with MK2206 or λ-phosphatase (Fig. 5F). Furthermore, when the threonine at T624 was substituted with aspartic acid (a phosphorylation-mimic mutant), overexpressing AKT and SKP2 diminished the levels of both ACSL4-WT and ACSL4-T624D, though to a lesser extent for the ACSL4-T624A mutant (Fig. 5G). The ACSL4-T624A mutation also disrupted the interaction between SKP2 and ACSL4 (Fig. 5H). Consistently, both SKP2 and AKT increased the ubiquitination of WT and ACSL4-T624D, but not of T624A (Fig. 5I), indicating that AKT-induced phosphorylation at T624 on ACSL4 enhances SKP2 recruitment, culminating in the ubiquitination of ACSL4 at the K621 site. Pursuing a deeper understanding of the regulatory mechanism within the LHPP/AKT-SKP2-ACSL4 axis, we first confirmed by western blot that LHPP knockdown significantly enhances SKP2-driven ACSL4 degradation relative to control, a process that MK2206 could inhibit (Fig. S6C). Additionally, LHPP knockdown also increased SKP2-mediated K63-linked multi-ubiquitin degradation of ACSL4 (Fig. 5J). Regarding cellular function, the reintroduction of LHPP into LHPP-knockdown cells substantially promoted Erastin-induced cell death and lipid peroxidation compared to treatments with SC79, overexpression of SKP2, or ACSL4 knockdown. This potentiation of cell death could be effectively counteracted by a ferroptosis inhibitor (Fig. S6D, E). Further, while AKT activation improved cell viability, SKP2 knockdown or ACSL4-WT overexpression significantly negated this viability, especially in the presence of overexpressed ACSL4-T624A (Fig. 5K and Fig. S6F). Likewise, overexpression of ACSL4-T624A markedly increased Erastin-induced lipid peroxidation (Fig. 5L and Fig. S6G). Collectively, our findings underscore the pivotal role of ACSL4-mediated lipid peroxidation and ferroptosis in mediating the tumor-suppressive activity of LHPP.

### Effectiveness of Panobinostat in upregulating LHPP expression via HDAC3 inhibition in PCa

**Fig. 6:**
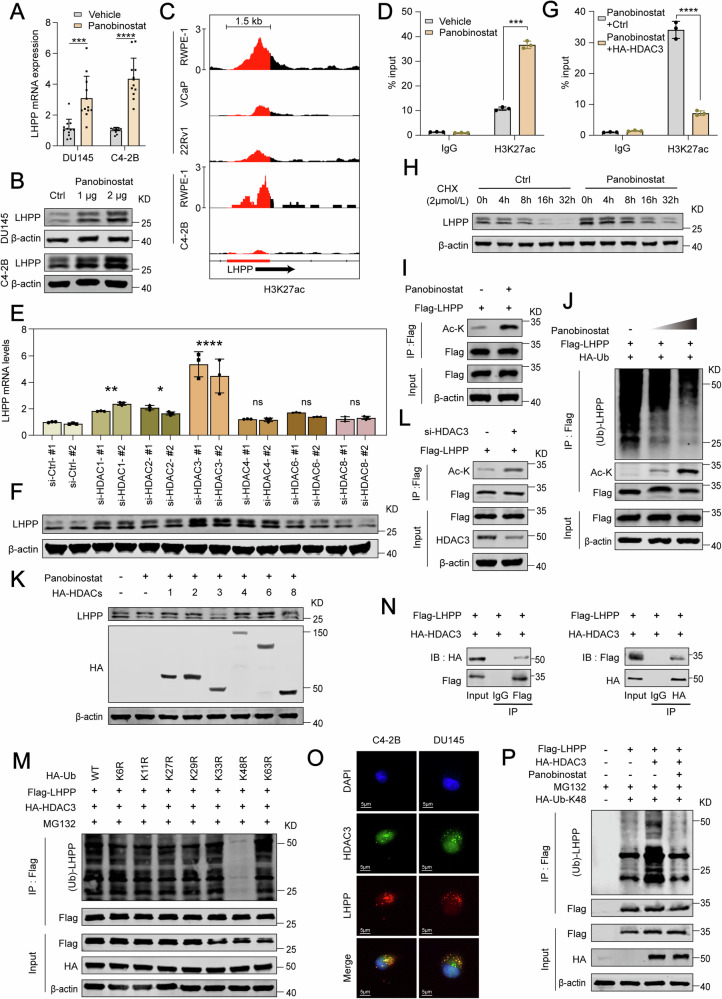
Panobinostat enhances LHPP expression through HDAC3 inhibition in PCa. **A**, **B** Significant increase in LHPP mRNA and protein expression in DU145 and C4-2B cell lines after Panobinostat (100 nM) treatment for 48 h. **C** Representative ChIP-seq tracks for H3K27ac on the LHPP promoter and gene body region in normal prostate cell lines versus PCa cell lines. Datasets from GES105760, GSE105290, GSE105627, and GSE78913. **D** ChIP-qPCR analysis of H3K27ac enrichment at the LHPP promoter after Panobinostat (100 nM) treatment for 24 h in DU145 cells. **E**, **F** The impact on LHPP expression through RT-qPCR and Western blot analysis, following the siRNA-mediated silencing of HDACs. **G** ChIP-qPCR analysis of H3K27ac enrichment at the LHPP promoter with the following Panobinostat treatment in DU145 cells with overexpressed HDAC3. **H** Western blot analysis of the significant upregulation in LHPP protein half-life following Panobinostat (100 nM) treatment followed by treatment with CHX (2 μM) for 0, 4, 8, 16, or 36 h. **I** The acetylation levels of Flag-tagged LHPP in DU145 cells were detected by Co-IP following the treatment with Panobinostat (100 nM) for 24 h. **J** Western blot analysis of ubiquitination levels of Flag-tagged LHPP after treatment with Panobinostat (0, 50, 100 nM) for 24 h. **K** Western blot analysis of LHPP protein expression in PCa cells with or without Panobinostat treatment (100 nM) following the indicated HDACs overexpression. **L** The acetylation levels of Flag-tagged LHPP in DU145 cells were detected by Co-IP following the knockdown of HDAC3. **M** Western blot analysis was used to assess changes in Flag-LHPP ubiquitination levels following ectopic transfection with HDAC3 and various lysine mutant forms of ubiquitin. **N** Western blot analysis of the interaction between Flag-tagged LHPP and HA-tagged HDAC3 revealed by Co-IP experiments. **O** Co-localization of LHPP and HDAC3 in DU145 and C4-2B cells. **P** Western blot analysis of the ubiquitination of Flag-tagged LHPP transfected with HA-tagged HDAC3 with or without Panobinostat treatment (100 nM) for 24 h. Statistical significance was determined by unpaired t-test (**A**, **D**, **G**) or Two-way ANOVA (**E**) and data are represented as mean ± SD. * *p* < 0.05; **p < 0.01; ***p < 0.001; ****p < 0.0001, respectively.

Our research data indicate that LHPP is underexpressed in PCa cells and acts as a tumor suppressor, which could significantly inhibit the progression of PCa cells. With the advancement of precision medicine, we aim to pinpoint pharmacological agents that could enhance treatment for patients with low LHPP expression. To this end, we utilized the CTRPv2 and PRISM public databases to investigate the relationship between LHPP expression and drug response, focusing on drug susceptibility and gene expression in cell lines, as depicted in Fig. S7A. Given that PCa is a solid tumor, we excluded non-tumor hematopoietic cell lines and those of unspecified origin, along with compounds possessing a non-applicable (NA) ratio above 25%. This screening yielded 394 drugs across 690 cell lines (CCLs) from CTRPv2, and 861 drugs across 482 CCLs from PRISM. Solid tumors were categorized based on LHPP expression levels, and Area Under the Curve (AUC) values were analyzed for differences using a Wilcoxon test. From both datasets, the top 20 compounds with a negative Log2FC (Fold change) differential were selected as differentially responsive drugs. These were further screened for compounds where the AUC value exhibited a negative correlation with the LHPP risk score (*R* > 0.1). Consequently, we identified 18 drugs from the CTRPv2 dataset and 15 from the PRISM dataset, respectively (Fig. S7B, C). Many of these drugs shared pharmacological properties, notably as histone deacetylase inhibitors (HDACi) and mitogen-activated protein kinase inhibitors (MEKi) (Fig. S7D, E). Consequently, based on an analysis that grouped drugs with similar effects, six drugs from CTRPv2 and four from PRISM were selected for further study. These drugs demonstrated promising sensitivity, however, their clinical relevance in treating prostate cancer required further validation. We embarked on a comprehensive search for clinical studies and experimental data related to these compounds in PCa treatment, using the PubMed database. We also utilized CMap data to construct and analyze compounds that contrasted with the PCa gene expression pattern and LHPP-specific differential gene expression pattern. The results revealed that the CMap scores for the compounds Merck60, Panobinostat, and Apicidin were below -2 (Fig. S7F, G). Therefore, based on clinical and experimental data, as well as CMap data predictions, we chose Panobinostat, Apicidin, and Merck60 these three compounds hold significant promise for treating PCa. In vitro validation in DU145 and C4-2B cell lines showed that only Panobinostat significantly increased LHPP mRNA and protein levels (Fig. 6A, B and Fig. S8A, B). Further investigations were conducted to unravel how Panobinostat regulates LHPP expression. The role of histone acetylation in epigenetic control is well-documented, influencing gene expression by modifying acetylation levels at gene promoters [33, 41]. A survey of ChIP-seq data, particularly the acetylation at LHPP transcriptional upstream regions, revealed a notable enhancement of H3K27ac in both normal and cancerous prostate cell lines (Fig. 6C). Additionally, ChIP-qPCR experiments confirmed the enrichments of H3K27ac at the LHPP TSS, suggesting a correlation with increased LHPP expression in PCa (Fig. S8C). Subsequent ChIP-qPCR experiments post-Panobinostat treatment corroborated a significant rise in H3K27ac levels at the LHPP promoter (Fig. 6D), and western blot analysis confirmed this finding (Fig. S8D). This suggests that the increase in H3K27ac due to Panobinostat correlates with the increased expression of LHPP in PCa. Given the broad-spectrum inhibitory activity of Panobinostat against the class I/II HDAC family, our study aimed to identify the specific HDAC member that modulates LHPP expression. We accordingly selected its target points of drug action for siRNA silencing tests (Fig. S8E), pinpointing HDAC3 as a significant regulator of LHPP expression at both the mRNA and protein levels (Fig. 6E, F). Subsequently, we induced the overexpression of various HDACs in PCa cell lines treated with Panobinostat to determine their individual roles in regulating LHPP expression. Notably, overexpression of HDAC3 led to a marked increase in H3K27ac accumulation at the LHPP TSS (Fig. 6G and Fig. S8F). As previously mentioned, we observed an increased presence of the LHPP protein following Panobinostat administration (Fig. 6B). To discern whether this elevation in protein levels resulted from increased mRNA synthesis or a post-translational mechanism, we conducted LHPP protein degradation half-life assays in prostate cancer cells treated with Panobinostat (Fig. 6H and Fig. S8G). These assays implied that Panobinostat may enhance LHPP expression not merely through transcriptional enhancement, but also by impeding its degradation. Significantly, acetylation and polyubiquitination can scrutinize the identical lysine residue, resulting in competitive binding [42]. Consequently, we investigated whether Panobinostat impacts the acetylation and ubiquitination of LHPP. Previous studies have indicated that HDAC4 fosters the ubiquitin-proteasome degradation of LHPP by restraining its acetylation level [34]. This led us to probe whether Panobinostat might influence LHPP stability through a similar mechanism. Post-treatment acetylation assessment indicated a substantial uptick in LHPP acetylation upon Panobinostat exposure (Fig. 6I). Subsequent analyses using Western blot to assess the correlation between LHPP acetylation and its protein stability uncovered a dose-dependent diminution in LHPP ubiquitination following Panobinostat administration (Fig. 6J). To clarify HDACs’ role in modulating LHPP protein levels post-Panobinostat treatment, we engineered the overexpression of different HDACs in prostate cancer cells. Intriguingly, only overexpression of HDAC3 significantly attenuated the Panobinostat-mediated increase in LHPP protein (Fig. 6K). Notably, the downregulation of HDAC3 led to enhanced LHPP acetylation (Fig. 6L). In contrast, overexpression of HDAC3, combined with MG132 treatment, increased LHPP expression, an effect not observed with chloroquine treatment (Fig. S8H). Furthermore, a significant decrease in K48R-linked ubiquitination of LHPP was observed with overexpressed HDAC3 (Fig. 6M), indicating that the proteasomal degradation of LHPP protein induced by HDAC3 occurs via the ubiquitin-proteasome pathway. Immunoprecipitation assays confirmed an interaction between LHPP and HDAC3 (Fig. 6N), corroborated by immunofluorescence co-localization within cells (Fig. 6O). Treatment with Panobinostat reduced the ubiquitination of Flag-tagged LHPP in cells overexpressing HDAC3 (Fig. 6P). These findings imply that Panobinostat may modulate LHPP levels by affecting its acetylation and subsequent ubiquitination, potentially via HDAC3. Furthermore, we utilized HDAC3 inhibitors such as RGFP966 to test their effects on LHPP, finding that treatment increased H3K27ac levels at the LHPP promoter (Fig. S8I) and significantly raised LHPP mRNA and protein levels, as confirmed by western blot and RT-qPCR analyses (Fig. S8J). Additionally, cell viability (Fig. S8K) and clonogenic assays (Fig. S8L, M) demonstrated a notable decrease in prostate cancer cell survival due to Panobinostat and RGFP966, although the inhibitory effect on cell proliferation was less pronounced with RGFP966 than with Panobinostat. This effect was mitigated when LHPP was silenced, indicating the dependency of these effects on LHPP presence. Notably, while RGFP966 hindered the accelerated proliferation of prostate cancer cells induced by LHPP deficiency, it did not perform as effectively as Panobinostat in this regard. Therefore, although various HDAC3 inhibitors can enhance LHPP expression and exert corresponding functions, their precise mechanisms may differ. In summary, our findings indicate that Panobinostat may serve as a promising therapeutic strategy for prostate cancer by inhibiting HDAC3, enhancing H3K27 acetylation in the LHPP promoter region to augment its transcription, and stabilizing LHPP protein through the modulation of its acetylation and ubiquitination.

### Panobinostat regulates LHPP and ACSL4-dependent ferroptosis to halt PCa progression

**Fig. 7:**
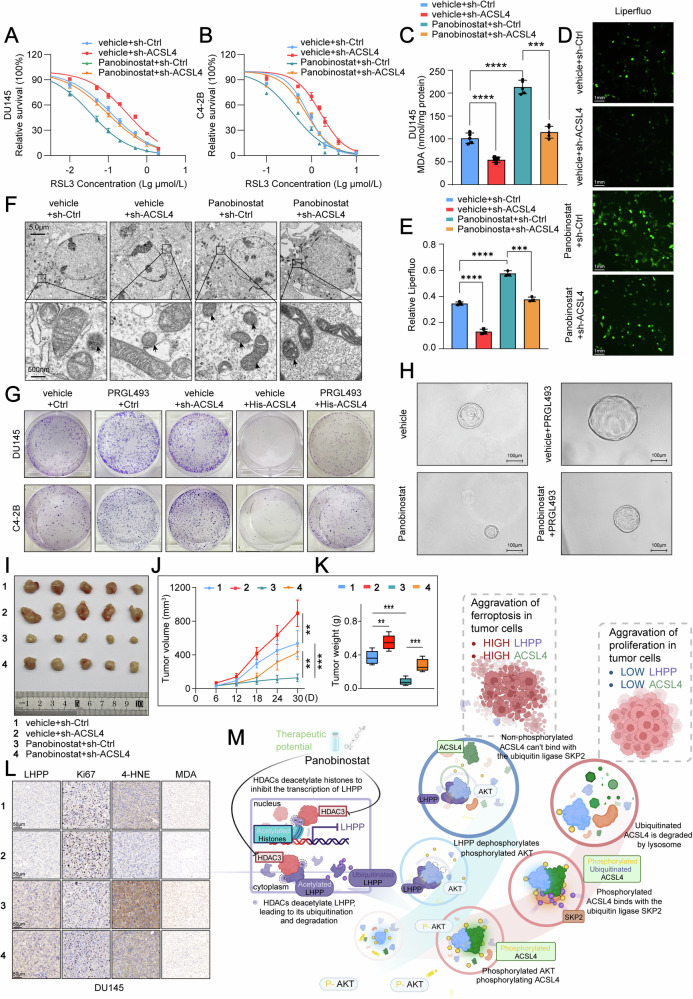
Panobinostat modulates LHPP and ACSL4-driven ferroptosis, contributing to the suppression of PCa development. **A**, **B** The sensitivity of RSL3 in DU145 and C4-2B cells treated with Panobinostat (100 nM) for 24 h compared to control and ACSL4-knockdown cells. **C** The levels of MDA in DU145 cells treated with Panobinostat (100 nM) for 24 h compared to control and ACSL4-knockdown cells. **D**, **E** The levels of Liperfluo in DU145 cells treated with Panobinostat (100 nM) for 24 h compared to control and ACSL4-knockdown cells. **F** The morphological changes of mitochondria were detected by transmission electron microscopy in DU145 cells treated with Panobinostat (100 nM) for 24 h compared to control and ACSL4-knockdown cells. **G** The colony formation assays of the effect of PRGL493 (5 μM) treatment in DU145 and C4-2B cells were modulated with or without ACSL4 knockdown and overexpression. **H** Organoid culture treated with Panobinostat and/or PRGL493 from PCa patient tissues. **I–K** Representative images illustrate in vivo experiments using a nude mouse model with subcutaneous tumors derived from the control DU145 cell line and ACSL4-knockdown DU145 cells, demonstrating the inhibitory effect of orally administered Panobinostat on tumor growth, and reversal of this effect with ACSL4 knockdown. The tumor growth (**I**), tumor volume (**J**), and tumor weight (**K**) were monitored. **L** Levels of IHC staining of LHPP, Ki67, MDA, and 4-HNE in the tumor model using DU145 cells. **M** Model for the role of an LHPP-AKT-ACSL4 axis in prostate tumorigenesis and ferroptosis. The model was created with BioRender.com. Statistical significance was determined by unpaired t-test (**C**, **E**, **K**) or One-way ANOVA (**J**) and data are represented as mean ± SD. **p < 0.01; ***p < 0.001; ****p < 0.0001, respectively.

Considering the pivotal role of ACSL4 in ferroptosis, orchestrated by LHPP, our research extended to examine the association between Panobinostat and ACSL4. Drawing from previous findings, we posited that the introduction of Panobinostat might trigger an accumulation of LHPP, which could regulate ferroptosis in an ACSL4-dependent manner. To confirm if Panobinostat enhances the efficacy of the ferroptosis inducer RSL3, PCa cells were exposed to Panobinostat. The results underscored a marked increase in RSL3 sensitivity in cells treated solely with Panobinostat, compared to untreated controls. However, this heightened sensitivity was significantly reduced when ACSL4-knockdown cells were co-treated with Panobinostat (Fig. 7A, B). Further, assays for MDA and Liperfluo (which recognizes lipid peroxides that are markers for ferroptosis activation) suggested that ACSL4 knockdown could reverse Panobinostat-induced ferroptosis (Fig. 7C–E and Fig. S9A). Subsequent electron microscopy revealed significant mitochondrial shrinkage in PCa cells under Panobinostat treatment; however, this effect was mitigated when ACSL4-knockdown cells were co-treated with Panobinostat (Fig. 7F). Reflecting on these results, we deduce that ACSL4 is essential for Panobinostat-induced ferroptosis. Furthermore, we explored whether RGFP966 shares similar functionalities with Panobinostat concerning LHPP and ACSL4. Upon initially treating cells with downregulated ACSL4 using both Panobinostat and RGFP966, we discovered that RGFP966 only partially restored the function of ACSL4 in curbing the proliferation of prostate cancer cells, unlike the significant restoration seen with Panobinostat (Fig. S9B–D). Moreover, assays for MDA and ROS indicated that RGFP966 only partially restored the capability of ACSL4 to promote lipid peroxidation and ferroptosis in prostate cancer cells (Fig. S9E, F). Consequently, when using the specific HDAC3 inhibitor RGFP966 to suppress HDAC3, its effects on ACSL4 were slightly less than those observed with Panobinostat. Besides, we noted that the inhibitory effects of Panobinostat on prostate cancer cells were superior to those of RGFP966. Given Panobinostat’s broader clinical application, compared to RGFP966 which is primarily used in scientific research and pre-clinical trials, we have opted for Panobinostat as the primary subject of our research on regulating LHPP-ACSL4. In order to assess Panobinostat’s impact on the in vivo growth of PCa, we engineered organotypic tissue cultures of prostate cancer. PRGL493, a potent and selective ACSL4 inhibitor, has been reported to attenuate ACSL4 activity [43]. Clonogenic and ROS assays were conducted to ascertain PRGL493’s functionality, mirroring ACSL4-knockdown effects. The results reveal that PRGL493 markedly increases the proliferation of DU145 and C4-2B cells and significantly decreases the generation and accumulation of ROS. Importantly, these inhibitory effects could be counteracted by the exogenous overexpression of ACSL4. Thus, we treated organoids with PRGL493 to replicate the knockdown effects of ACSL4 in vitro (Fig. 7G and Fig. S9G, H). When treated with Panobinostat, the growth of these organoids was significantly suppressed, yet this inhibitory effect could be counteracted by treatment with PRGL493 (Fig. 7H). Furthermore, we conducted validation using a nude mouse model. We established a subcutaneous tumor model in mice using the DU145 and C4-2B cell lines with an ACSL4 knockdown (Fig. S9I). Results indicated that oral administration of Panobinostat significantly impeded tumor growth, and this inhibition was reversed by ACSL4 knockdown (Fig. 7I–K and Fig. S9J–L). IHC staining was used to validate the activation of ferroptosis in vivo (Fig. 7L and Fig. S9M). We conclude that Panobinostat’s inhibitory influence on prostate cancer growth and progression is mediated through the upregulation of LHPP, closely associated with the ferroptosis-related gene ACSL4.

In conclusion, our research suggests that Panobinostat instigates an increase in LHPP, which in turn dampens AKT phosphorylation levels. This reduction lessens the interaction between ACSL4 and SKP2, resulting in elevated ACSL4 levels and promoting ferroptosis onset. Therefore, LHPP is implicated in mediating the ferroptosis process (Fig. 7M).

## Discussion

LHPP, a phosphatase identified as a tumor suppressor, is notably underexpressed in various human malignancies. This underexpression correlates with aggressive disease traits and a poor prognosis [8, 11, 34]. Despite its recognition as a tumor suppressor over the past five years [8], the underlying mechanisms by which LHPP influences oncogenesis remain largely obscured. Our study validates LHPP as a genuine suppressor of prostate cancer, with its deficiency linked to an unfavorable patient outcome, thereby positing therapeutic targeting of LHPP as a potential strategy to curb prostate cancer progression and dissemination. We also present substantial evidence that LHPP significantly boosts ferroptosis sensitivity in prostate cancer cells, suggesting that LHPP is a hitherto unidentified promoter of ferroptosis.

In our research, we have determined that LHPP deficiency leads to reduced expression of ACSL4, a key enzyme in PUFA phospholipid conversion and lipid peroxidation, both vital for ferroptosis susceptibility [17, 18]. Cellular metabolism, especially lipid metabolism, and protein degradation pathways appear to be integral to ferroptosis sensitivity [44]. Our results suggest that LHPP modulates ferroptosis through an ACSL4-dependent pathway, controlling ACSL4 phosphorylation and thus its protein stability. Notably, LHPP does not directly alter ACSL4’s phosphorylation status, hinting at an intermediary mechanism that governs ACSL4 regulation by LHPP. Several studies have shed light on the role of LHPP in inhibiting tumor growth via repression of AKT activation, evidenced in various cancers such as hepatocellular carcinoma [8], nasopharyngeal carcinoma [34], oral squamous cell carcinoma [10], and notably prostate cancer [6]. Nonetheless, the precise manner in which LHPP affects AKT activity and, consequently, prostate cancer prognosis, remains elusive. Earlier studies have indicated that AKT activation downregulates ACSL4 [27], suggesting a potential liaison between AKT and ACSL4. The intricacies of LHPP’s influence on AKT activation and AKT’s impact on ACSL4 are yet to be clarified, warranting further investigation given the clinical significance of LHPP-induced ferroptosis.

Our study demonstrates that LHPP binds to AKT, inhibiting its activation and thereby impacting various cellular functions. A critical discovery is ACSL4’s identification as a novel phosphorylation target for AKT, which promotes ACSL4 degradation via phosphorylation. The literature underscores a strong link between phosphorylation and ubiquitination, both pivotal in protein interaction regulation and tumor progression [45]. Phosphorylation often precedes recognition by the F-Box component of SCF ligase in substrate proteins [23], with several tumor-associated proteins known to undergo AKT-mediated phosphorylation and ubiquitination [24, 46, 47]. We demonstrate that AKT modulates ACSL4 by phosphorylating the T624 site, which activates ACSL4 to interact with SKP2, leading to its K63-linked polyubiquitination and subsequent proteasomal degradation. By inhibiting AKT activation, we prevent SKP2 from recognizing the K621 site on ACSL4, a conclusion that is reinforced by the outcomes observed after knocking down SKP2. This indicates that AKT-induced phosphorylation is essential for ACSL4 degradation by SKP2.

The existing body of research acknowledges the critical role of ferroptosis in human cancers [48–50], with drug-resistant cancer cells exhibiting increased vulnerability to GPX4 inhibitors in vitro [51]. This positions pharmacologically induced ferroptosis as a promising anticancer approach. Our study underscored the pan-HDAC inhibitor effect of Panobinostat on reversing LHPP underexpression in prostate cancer cells. HDAC inhibitors, including Panobinostat, SAHA, and Romidepsin, are extensively studied in prostate cancer treatment, with these agents currently undergoing clinical trials [29, 52, 53]. While further research is needed to clarify Panobinostat’s specific effectiveness in prostate cancer therapy, preliminary laboratory and clinical studies suggest its therapeutic potential. Our research indicates Panobinostat as a prospective agent to target LHPP, enhancing H3K27ac levels at LHPP’s transcription start site, thus increasing LHPP’s transcription and expression. Acetylation’s role in regulating both histone and non-histone proteins is well-documented, yet understanding of LHPP as an acetylation-modified non-histone protein is limited. Identifying the optimal usage strategy for Panobinostat, including its side effects and interactions with other treatments, is critical for future research. Furthermore, we discovered LHPP not only a PTM (Post-Translational Modification) substrate of HDAC3 but also as a novel histone target for HDAC3, with HDAC3-mediated reduction of H3K27ac levels at LHPP’s transcription start site inhibiting its transcriptional activation. This highlights the complex regulatory mechanisms governing LHPP expression and stability, which are crucial for maintaining its tumor-suppressive function.

Our study acknowledges certain limitations, including the need for further research to delineate Panobinostat’s precise role in the LHPP protein degradation process. Moreover, the extrapolation of results from cell lines and animal models to human physiology necessitates validation through clinical trials to ascertain the drug’s safety and efficacy in the treatment of prostate adenocarcinoma.

In conclusion, our study underscores LHPP as a suppressor of prostate cancer. Mechanistically, the low expression of LHPP leads to the abnormal activation of AKT, which promotes the binding of AKT with ACSL4. The activated ACSL4 recruits SKP2, which mediates its degradation via the ubiquitin-lysosome pathway. Additionally, our data reveal new roles for Panobinostat and HDAC3 in the epigenetic regulation of LHPP. The drug enhances the H3K27ac level at LHPP’s transcriptional start site, thereby activating LHPP transcription, boosting LHPP expression, and, in turn, regulating ACSL4 activity, ultimately inducing ferroptosis. In summary, our research offers convincing evidence that LHPP is a promising biomarker and therapeutic target in prostate cancer.

## Materials and methods

### Cell lines and cell culture

The HEK-293T, DU145, and C4-2B cell lines were acquired from the esteemed American type culture collection (ATCC). The C4-2B and DU145 lines were cultivated in an RPMI-1640 medium (Pricella Life Science & Technology Co., Ltd), whilst the 293T line was grown in a DMEM medium (Cat # BMC1010, Abbkine, Wuhan, China). All cell cultures were consistently kept at a temperature of 37 °C and a CO_2_ concentration of 5%, with each medium further supplemented with 10% fetal bovine serum.

### Reagents, plasmids, siRNA, and shRNA

The chemical compounds, including Panobinostat (LBH589) (HY-10224), Erastin (HY-15763), RSL3 (HY-100218A), Apicidin (HY-N6735), MG132 (HY-13259), PRGL493 (HY-139180), NH_4_Cl (HY-Y1269), 3-methyladenine (3-MA) (HY-19312), Bafilomycin A1 (BafA1) (HY-100558), and Cycloheximide (CHX) (HY-12320) were supplied by MCE (Shanghai, China). MK2206 (SJ-MX0056) and RGFP966 (SJ-MX0358) were supplied by Sparkjade (Shandong Sparkjade Biotechnology Co., Ltd). SC79 (T2274) and Ferrostatin-1 (Fer-1) (T6500) were obtained from Topscience, and Merck60 (H2595) from Hmobio, both companies based in Shanghai, China.

Plasmids of Flag-LHPP, HA-Ub, Myc-AKT1, His-ACSL4, Flag-CD44, Flag-NDRG1, Flag-SKP2, and HA-HDAC3 were synthesized and purchased from the Public Protein/Plasmid Library in Jiangsu, China. The shRNAs targeting LHPP and ACSL4 vectors were provided by Hanbio (Shanghai, China), and the siRNA constructs were sourced from Ribo Biotechnology (Guangzhou, China). The specific sequences targeted by these shRNAs and siRNAs are detailed in Supplementary Table S2.

### Transfection and lentivirus infection

Cells were transfected with plasmid DNA using Lipofectamine 3000 (Thermo Fisher Scientific), and siRNA was introduced using the jetPRIME® Versatile DNA/siRNA transfection reagent (Polyplus Transfection), following the manufacturer’s instructions. Viral packaging was commenced in the HEK-293T cells after co-transfection of the plasmid with the packaging plasmid psPAX2 and envelope plasmid

pMD2.G, facilitated by Lipofectamine 3000. Viruses were gathered approximately two days after the transfection. The target cells were subsequently exposed to the recombinant lentivirus-transducing units, alongside 8 μg/ml Polybrene (Beyotime).

### Human samples

Primary malignant tissue samples, alongside their respective adjacent normal tissues, were obtained with the appropriate approval from the Research Ethics Board (2024-KY-0092), with patients giving written informed consent. These samples originated from the First Affiliated Hospital of Zhengzhou University, Henan, China. LHPP mRNA levels were quantified by RT-qPCR analysis, while protein concentrations were ascertained using the Western blotting procedure.

### Western blot

Both cultured cells and homogenized human tissue samples were lysed using RIPA lysis buffer (cat. No. R0010; Beijing Solarbio Science & Technology Co., Ltd), supplemented with protease and phosphatase inhibitors (Epizyme Pharmaceutical Biotechnology). The protein concentration was measured using a BCA protein assay kit (Epizyme Pharmaceutical Biotechnology). Proteins were subsequently separated on 10% precast gradient gels (Epizyme Pharmaceutical Biotechnology) and transferred onto nitrocellulose membranes. After the transfer, the membranes were blocked with 5% non-fat milk in Tris-buffered saline with 0.05% Tween-20 (TBST). The membranes were then incubated overnight at 4 °C with the primary antibody, followed by a 1-h incubation at room temperature with either DyLight 800-Goat Anti-Rabbit IgG (1:100, A23910 Abbkine) or DyLight 800-Goat Anti-Mouse IgG secondary antibodies (1:100, A23920 Abbkine). Following this, the membranes were scanned using an imaging system (ODYSSEY CLx, Gene Company Limited), and the optical density was analyzed with Image Studio Lite (LI-COR Biosciences). The primary antibodies employed included those against LHPP (1:500, Proteintech, 15759-1-AP), Beta Actin (1:10000, Proteintech, 81115-1-RR), ACSL4 (1:2000, Proteintech, 22401-1-AP), CD44 (1:2000, Proteintech, 15675-1-AP), HMOX1 (1:2000, Proteintech, 10701-1-AP), NDRG1 (1:1000, Cell Signaling Technology, 5196), Flag (1:2000, Abbkine, ABT2010), Flag (1:2000, Proteintech, 20543-1-AP), HA (1:2000, Abbkine, ABT2040), HA (1:2000, Proteintech, 81290-1-RR), His (1:2000, Abbkine, ABT2050), His (1:2000, Proteintech, 25940-1-AP), p-AKT (1:500, Cell Signaling Technology, 4060), AKT (1:1000, Cell Signaling Technology, 4691), Myc (1: 1000, Santa Cruz, sc-40), Myc (1: 2000, Proteintech, 16286-1-AP), ATG7 (1:1000, Cell Signaling Technology, 8558), SKP2 (1:1000, Santa Cruz, sc-74477), H3K27ac (1:500, Abcam, ab4729), H3 (1:2000, Abcam, ab1791), and HDAC3 (1:1000, Proteintech, 10255-1-AP). Cropped blots are provided in the Source Data file.

### Immunohistochemistry

Tumor specimens were subjected to a 4% paraformaldehyde solution in PBS, swiftly embedded in an OCT compound, and meticulously sectioned into 6 μm slices. The pertinent antibody was applied in immunohistochemical analysis to illuminate cellular structures. Images were procured using a Leica DM IRB microscope (Wetzlar, Germany) and interpreted via the Image Pro Plus software. The primary antibodies employed for Immunohistochemistry comprised LHPP (1:200, Proteintech, 15759-1-AP), ACSL4 (1:200, Proteintech, 22401-1-AP), p-AKT (1:200, Cell Signaling Technology, 4060), and Ki67 (1:200, Servicebio, GB121141-100).

### Co-immunoprecipitation

Cellular lysis was facilitated with an IP lysis buffer (Servicebio, G2038), supplemented with protease and phosphatase inhibitors. To achieve pre-clearance, protein A/G magnetic beads (HY-K0202, MCE) were amalgamated with the protein lysate. The respective antibody was then integrated and incubated at 4 °C for a duration of two hours. This was followed by the addition of protein A/G magnetic beads and an overnight incubation at 4 °C. Thereafter, the beads were boiled in SDS loading buffer in anticipation of subsequent western blot analysis. The primary antibodies employed for immunoprecipitation comprised Ub (1: 500, Santa Cruz, sc-8017), Myc (1: 200, Santa Cruz, sc-40), Flag (1:200, Abbkine, ABT2010), HA (1:200, Abbkine, ABT2040), His (1:200, Abbkine, ABT2050), ACSL4 (1:100, Proteintech, 22401-1-AP), AKT (1:100, Cell Signaling Technology, 4691), SKP2 (1:100, Santa Cruze, sc-74477), control mouse IgG (1:200, Santa Cruz, sc-2025), control rabbit IgG (1:200, Proteintech, 30000-0-AP) and pan Phospho-Serine/Threonine (1:200, Beyotime, AF5725).

### GST pull-down

We constructed plasmids using the pGEX-4T-1 vector, which were expressed in E. coli BL21 (DE3) cells. These cells were plated on LB agar with ampicillin and incubated overnight at 37 °C. A single colony was expanded in LB broth with ampicillin, first in a 10 ml culture and then scaled up to 500 ml until reaching an OD600 of 0.6 at 25 °C. Induction was carried out with 0.5 mM IPTG at 16 °C for over 15 h. Cells were collected by centrifugation at 3000 rpm for 20 min at 4 °C, resuspended in cold PBS, and lysed in buffer containing PMSF and protease inhibitor cocktail, maintaining all procedures at 4 °C. After ultrasonication and centrifugation at 12,000 rpm for 30 min, the clear supernatant was retrieved. The lysate was incubated with GST Purification Magbeads (Absin, abs9902) for 2 h and washed with GST wash buffer (BJBiolab, GS4655) with PMSF. Protein interactions with His-ACSAL4 were captured overnight at 4 °C on the beads, washed, and eluted by boiling in an SDS loading buffer. The GST pull-down products were analyzed by Western blot.

### RNA isolation and qPCR

The SevenFast® Total RNA Extraction Kit (SM132-02, Seven/Abcells, Beijing, China) was utilized to isolate total RNA, adhering to the manufacturer’s specifications. The NovoScript®Plus All-in-one 1st Strand cDNA Synthesis SuperMix (E047-01A, Novoprotein, Shanghai, China) was used for the synthesis of cDNA, which was subsequently used for real-time PCR with the NovoStart® SYBR qPCR SuperMix Plus (E096-01A, Novoprotein) on a QuantStudio Three Real-Time PCR System (Thermo Fisher). Internal controls comprised β-actin or GAPDH. The qPCR primers are detailed in Supplementary Table S2.

### Lipid peroxidation assay

Cells were seeded in 6-well plates and cultured for 12 h to promote exponential growth. Subsequently, each well was refreshed with a serum-free medium containing 10 μM DCFH-DA (S0033S, Beyotime) for the detection of reactive oxygen species (ROS). Following a 20-min incubation at 37 °C in a 5% CO_2_ atmosphere, the medium was discarded, and the cells were washed three times with serum-free medium. Then, 1 ml of serum-free medium was added to each well. For lipid peroxidation analysis using Liperfluo, cells were incubated with 5 μM Liperfluo (Dojindo, Shanghai, China) for 30 min at 37 °C, following the manufacturer’s protocol. Post-incubation, the Liperfluo solution was removed, and the cells were washed three times with Hank’s Balanced Salt Solution (HBSS). Fluorescence microscopy (Leica DM IRB, Wetzlar, Germany) was employed to capture the images of the stained cells.

### Immunofluorescence staining

Cells were seeded in 6-well plates containing a circle (801007, Nest, Wuxi, China) and cultured for 24 h. The primary antibody was incubated overnight at 4 °C, followed by a one-hour secondary antibody incubation. Microscopic images were procured using Leica DM IRB microscopes (Wetzlar, Germany). The primary antibodies employed for immunofluorescence comprised ACSL4 (1:200, Proteintech, 22401-1-AP), AKT (1:300, Cell Signaling Technology, 4691), LAMP2 (1:200, Cell Signaling Technology, 34141) and HDAC3 (1:200, Proteintech, 10255-1-AP).

### Transmission electron microscopy

Cells were centrifuged into clusters, then fixed with a 2.5% glutaraldehyde-phosphate buffer solution for 2 h, and rinsed thrice with 0.1 M phosphate rinse solution for 15 min each. A 1% osmium acid solution was used for an additional fixation period of 2–3 h, followed by three rinses with 0.1 M phosphate rinse solution for 15 min each. After being dehydrated with 50%, 70%, and 90% ethanol, cells were embedded and fixed in acetone, then sectioned (70 nm) (Leica EM KMR3). Following a double-stain with 3% uranyl acetate-lead citrate, cells were examined using a transmission electron microscope (JEM-1230), and images were obtained.

### ChIP-qPCR

ChIP-qPCR analysis was performed on DU145 cells, adhering to the instructions provided by the SimpleChIP® Enzymatic Chromatin IP Kit (Magnet Beads) (9003, Cell Signaling Technology). We cross-linked 5 × 10^6^ cells with 1% formaldehyde for 10 min at room temperature, halting the reaction with 2 ml of 10× glycine for 5 min. Cells were then washed with PBS, lysed in cold Buffer A supplemented with DTT and PIC, and incubated on ice for 10 min. Post-centrifugation, the nuclear pellets were digested with micrococcal nuclease in Buffer B containing DTT at 37 °C for 20 min, with the reaction quenched by 50 mM EDTA. The nuclear fractions were then sonicated to fragment the nucleosomes, using a Xinzhi sonicator with a 1/8-inch probe for three 20-s intervals. A 2% aliquot of this sonicated material was set aside as an input control. Antibodies targeting H3K27ac (ab4729, Abcam), H3 (ab1791, Abcam), and IgG isotype control (3900, Cell Signaling Technology) were then introduced to the sonicated chromatin and incubated overnight at 4 °C. Upon addition of Protein G Magnetic Beads, the mixtures were incubated for an additional two hours at 4 °C. Following magnetic separation and successive washes, DNA was eluted from the beads, and cross-links were reversed at 65 °C in the presence of Proteinase K. The DNA was then purified using spin columns and quantified by qPCR to evaluate enrichment.

### Organoid culture

Human prostate cancer tissues acquired from the First Affiliated Hospital of Zhengzhou University were meticulously minced into fine fragments (1–5 mm^3^) using a scalpel in a sterile 10 cm culture dish. These fragments were then incubated at 37 °C overnight in a 15 ml conical tube containing a solution of collagenase II (5 mg/ml) and Y-27632 (10 µM), on an orbital shaker. It is advisable to use 1 ml of the collagenase II solution for each 50 mg of tissue. Post-digestion, the tissues were rinsed by adding F12K medium to reach a total volume of 10 ml, followed by centrifugation at 200 × *g* for 5 min at 4 °C. The sedimented cells were resuspended in 1 ml of TrypLE, supplemented with 10 µM Y-27632, and incubated for an additional 15 min at 37 °C to enhance digestion. After this secondary digestion, the cell pellet was washed by filling with F12 medium to a 10 ml volume and centrifuged at 200 × *g* for 5 min at 4 °C. The supernatant was carefully aspirated, and the digested tissue was then embedded in ice-cold Matrigel, ensuring homogeneity by pipetting the mixture 5–10 times. Using a hemocytometer, the cell count was determined, and approximately 20,000 cells were seeded in a 40 µl droplet at the center of each well of a 24-well plate. The plate was then placed in a 37 °C incubator for 15 min to permit the Matrigel to polymerize. Thereafter, 500 µl of pre-warmed (37 °C) human prostate culture medium, also supplemented with 10 µM Y-27632, was gently added to each well. The medium was refreshed every 2–3 days, and after a period of 7 days, Y-27632 supplementation was ceased.

### In vivo tumor xenograft model

SCID mice were procured from the Sipeifu Company (Beijing, China). In a preliminary study, male BALB/c nude mice, 4–6 weeks of age, were randomly divided into two experimental groups. Each mouse was administered a subcutaneous injection of approximately 1 × 10^6^ DU145-sh-Ctrl or DU145-sh-LHPP cells, suspended in a 1:1 mixture of Matrigel (Becton, Dickinson and Company, USA), between the shoulder blades and the hind leg. Tumor sizes were measured every six days following the injection using the formula V = L × W^2^/2, where “L” is the longitudinal diameter and “W” is the transverse diameter. On day 30 post-inoculation, the mice were euthanized via cervical dislocation. The excised tumors were photographed, weighed, and fixed in 4% paraformaldehyde in preparation for immunohistochemical analysis. In a follow-up study, another set of nude mice received subcutaneous injections with 1 × 10^6^ DU145-sh-Ctrl or DU145-sh-ACSL4 stable cells. Beginning on the 6th day and continuing on the 12th, 18th, and 24th days post-injection, the mice were treated with either normal saline or Panobinostat. Tumor development was monitored at six-day intervals using the same volumetric formula. Thirty days after the initial injections, these mice were also humanely euthanized, and the tumors were collected, imaged, weighed, and fixed for subsequent immunohistochemical staining.

### TCGA and computational data analysis

In our pursuit to dissect the disparities in LHPP mRNA concentrations between standard and neoplastic tissues, we deployed RNA-seq data pertaining to prostate cancer from The Cancer Genome Atlas (TCGA), obtainable via the UCSC Xena browser. The H3K27ac ChIP-seq data pertinent to LHPP were procured from UCSC. To delve into the associations connecting gene expression and the response to therapeutics across an array of cancer cell lines, we employed resources from the Cancer Therapeutics Response Portal (CTRP) and the Pharmacological RNAi Screening in Mammals for Drug Repurposing (PRISM). The expression metric for the genes in the cell lines from CTRP and PRISM was derived from the data portal of the Cancer Cell Line Encyclopedia (CCLE).

### Statistical analysis

All experiments were repeated at least three times, and data are presented as mean ± standard deviation (SD). Comparisons between two groups are analyzed using an unpaired t-test, while comparisons among multiple groups were performed with one-way or two-way ANOVA, followed by Tukey’s post hoc test where appropriate. A *p*-value of <0.05 was considered statistically significant. All statistical analyses were conducted using SPSS software version 22.0 (IBM Corp, Armonk, NY, USA).

## Supplementary information

Supplementary Information

Unedited blot and gel images

## Data Availability

The datasets used and/or analyzed during the current study are available from the corresponding author upon reasonable request.
