# Supplementary material for: Phosphatase LHPP confers prostate cancer ferroptosis activation by modulating the AKT-SKP2-ACSL4 pathway
Source: Cell Death Dis. 2024 Sep 11;15(9):665. doi: 10.1038/s41419-024-07007-8 (PMC11390745; doi:10.1038/s41419-024-07007-8)
Supplement: Supplementary file 1 — Supplementary Information [file 41419_2024_7007_MOESM1_ESM.docx]

**Phosphatase LHPP confers prostate cancer ferroptosis activation by modulating the AKT-SKP2-ACSL4 pathway**

Guoqing Xie, Ningyang Li, Keqiang Li, Yating Xu, Yu Zhang, Shun Cao, Budeng Huang, Ruoyang Liu, Peijie Zhou, Yafei Ding, Yinghui Ding, Jinjian Yang, Zhankui Jia, Zhenlin Huang

**Files included in the supplementary information:**

**Supplementary figure 1** **（Supplemental to Figure. 1）**Lower LHPP expression tied to prostate cancer progression and poor prognosis.

**Supplementary figure 2** **（Supplemental to Figure 1）**The role of LHPP in prostate cancer cell proliferation, migration, and invasion.

**Supplementary figure 3** **（Supplemental to Figure 2）**LHPP promotes ferroptosis in PCa via upregulation of ACSL4 expression.

**Supplementary figure 4** **（Supplemental to Figure 2-3）**LHPP modulates ACSL4 protein expression indirectly by influencing AKT phosphorylation and the ubiquitin-lysosome pathway.

**Supplementary figure 5** **（Supplemental to Figure 4）**Role of SKP2 in LHPP/AKT-mediated ACSL4 degradation.

**Supplementary figure 6** **（Supplemental to Figure 5）**LHPP inhibits PCa proliferation by blocking AKT/SKP2-induced ACSL4 degradation to promote ferroptosis.

**Supplementary figure 7** **（Supplemental to Figure 6）**Potential therapeutic application of Panobinostat in PCa treatment.

**Supplementary figure 8** **（Supplemental to Figure 6）**Panobinostat enhances LHPP expression through HDAC3 inhibition in PCa.

**Supplementary figure 9** **（Supplemental to Figure 7）**Panobinostat regulates LHPP and ACSL4-dependent ferroptosis to halt prostate cancer progression.

**Supplementary Table S1**. Clinical characteristics of PRAD patients.

**Supplementary Table S2**. Primer shRNA and siRNA sequence information.

**Supplementary materials and methods**

**Supplementary figures**

**
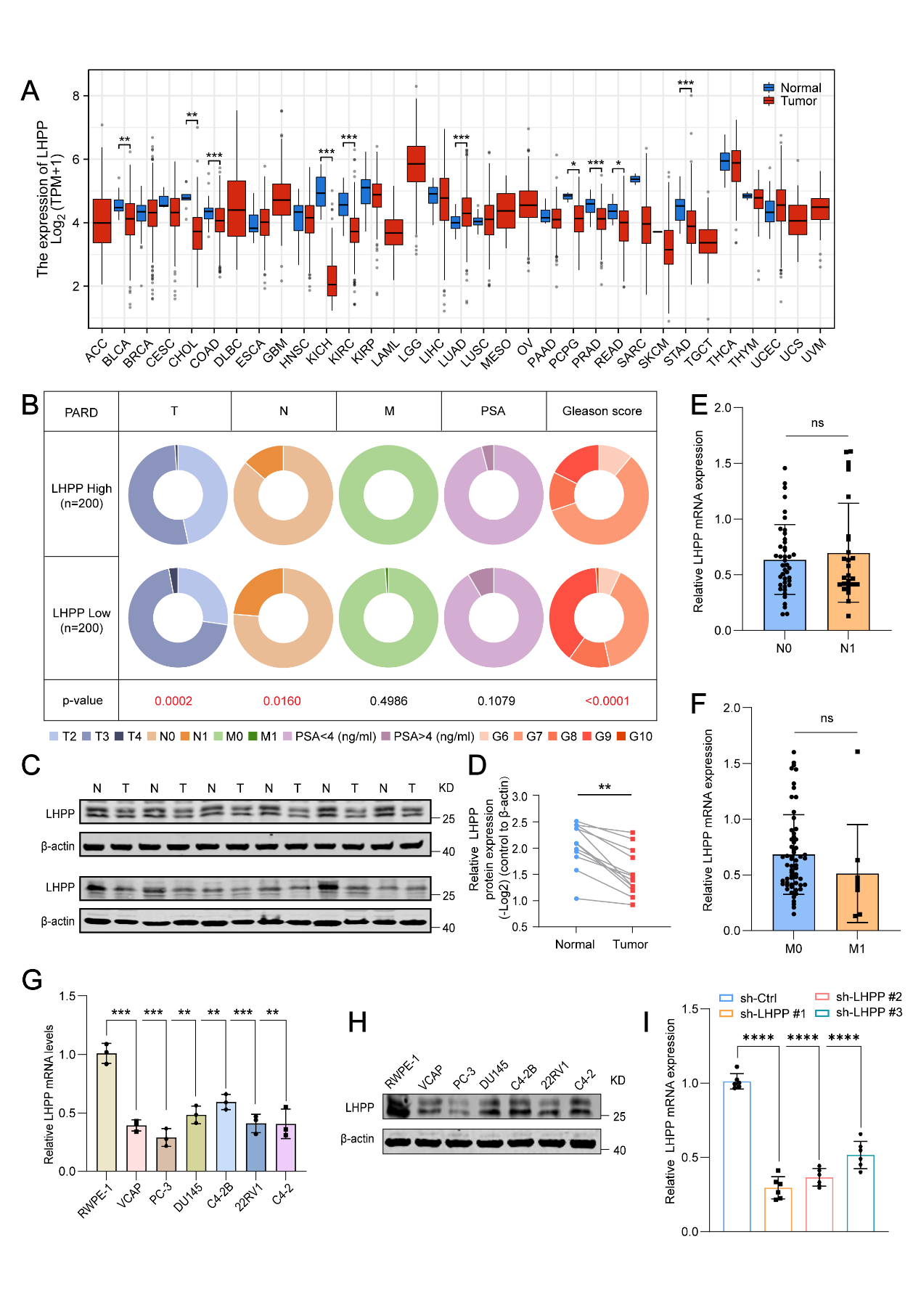
Supplementary figure 1. Lower LHPP expression tied to prostate cancer progression and poor prognosis.**

**(A)** LHPP expression levels in different tumor types from the TCGA database. **(B)** The pie chart delineates the variation in clinical indices, including T, N, M stages, PSA content, and Gleason score, between the LHPP-high and -low expression cohorts within the TCGA PRAD dataset. Statistical significance was determined by Chi-square and Fisher’s exact test. **(C-D)** Western blot assays indicating lower LHPP expression in prostate cancer tissues compared to adjacent tissues. **(E-F)** The correlation of LHPP expression with the N and M stage of prostate cancer was assessed through RT-qPCR in 70 prostate cancer patients. **(G-H)** LHPP expression in various prostate cancer cell lines. **(I)** LHPP expression in mRNA after transfection with three shRNAs in DU145 cells. Statistical significance was determined by unpaired t test **(A, D, E, F, G, I)** or Chi-square and Fisher’s exact test **(B)** and data are represented as mean ± SD. ns, not significant; * *p* < 0.01; ** *p* < 0.01; *** *p* < 0.001; **** *p* < 0.0001, respectively.


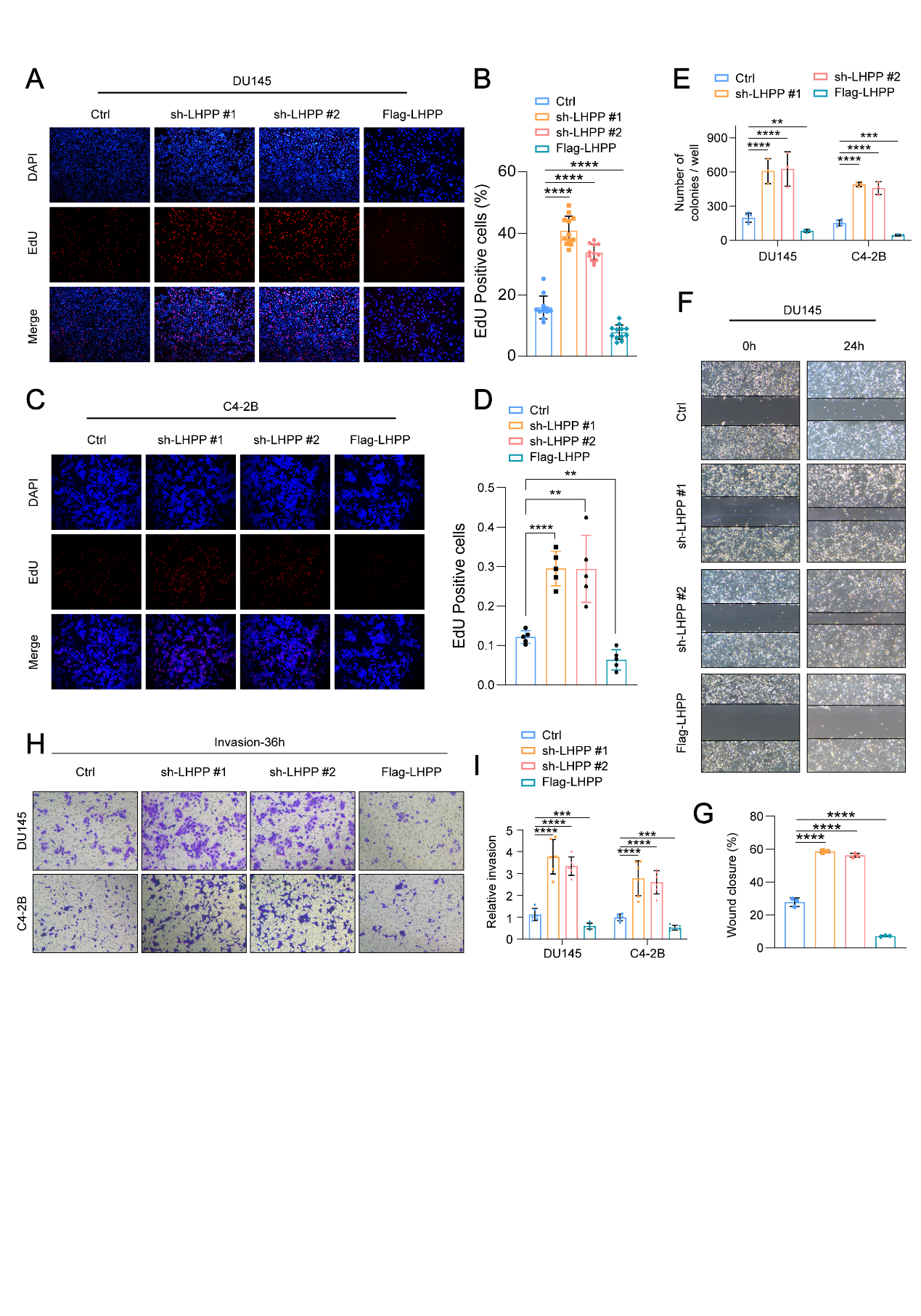


**Supplementary figure 2. The role of LHPP in prostate cancer cell proliferation, migration, and invasion.**

**(A-B)** Count of live, EdU-labelled DU145 cells and C4-2B **(C-D)** after LHPP knockdown and overexpression. **(E)** Colony formation assays in DU145 and C4-2B cells conducted to inspect cell proliferation. **(F-G)** Scratch assays in DU145 cells utilized for evaluating cell migration capability, and **(H-I)** transwell assays performed to analyze cell invasion ability, all subsequent to LHPP knockdown and over expression. Statistical significance was determined by unpaired t test **(B, D, E, G, I)** and data are represented as mean ± SD. ** *p* < 0.01; *** *p* < 0.001; **** *p* < 0.0001, respectively.


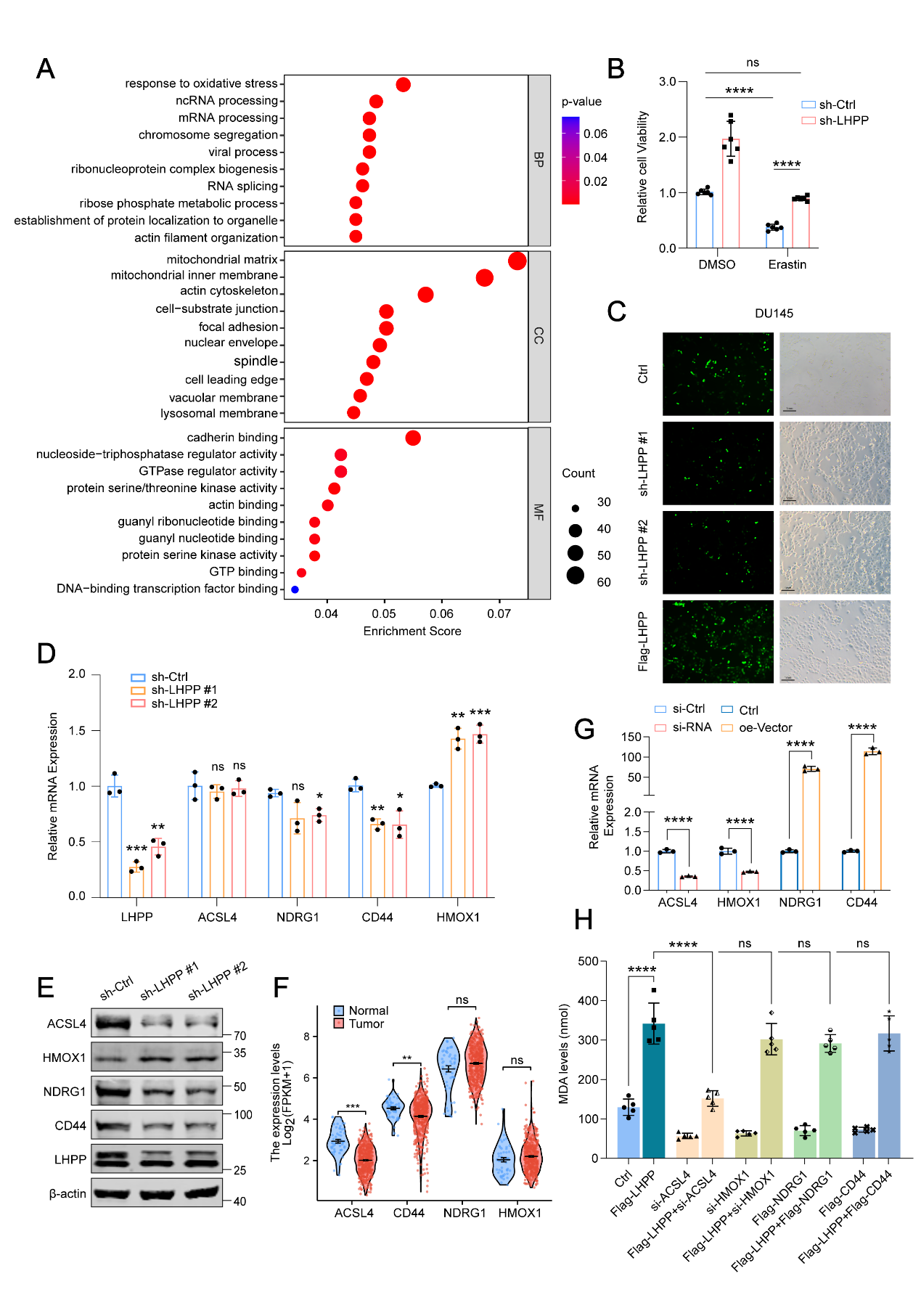


**Supplementary figure 3. LHPP promotes ferroptosis in PCa via upregulation of ACSL4 expression.**

**(A)** The GO analysis of Label-Free MS assays in DU145 cells after knockdown of LHPP. **(B)** Cell viability analysis following Erastin treatment (1 μM) for 24 h in control and LHPP-overexpression DU145 cells. **(C)** Representative images showing ROS in the DU145 cells after LHPP knockdown or overexpression. **(D)** RT-qPCR analysis of LHPP, ACSL4, NDRG1, CD44, and HMOX1 expression after LHPP knockdown. **(E)** Protein expression levels of ACSL4, NDRG1, CD44, and HMOX1 after LHPP knockdown. **(F)** Expression levels of ACSL4, NDRG1, CD44, and HMOX1 in PRAD within the TCGA database. **(G)** Expression levels of ACSL4, NDRG1, CD44, and HMOX1 in DU145 cells after transfection with siRNA or overexpression vector. **(H)** The levels of MDA in DU145 cells lines after overexpression of LHPP compared to control and ACSL4 and HMOX1 knockdown, or NDRG1and CD44 overexpression.

Statistical significance was determined by unpaired t test **(B, D, F, G, H)** and data are represented as mean ± SD. ns, not significant; * *p* < 0.05; ** *p* < 0.01; *** *p* < 0.001; **** *p* < 0.0001, respectively.


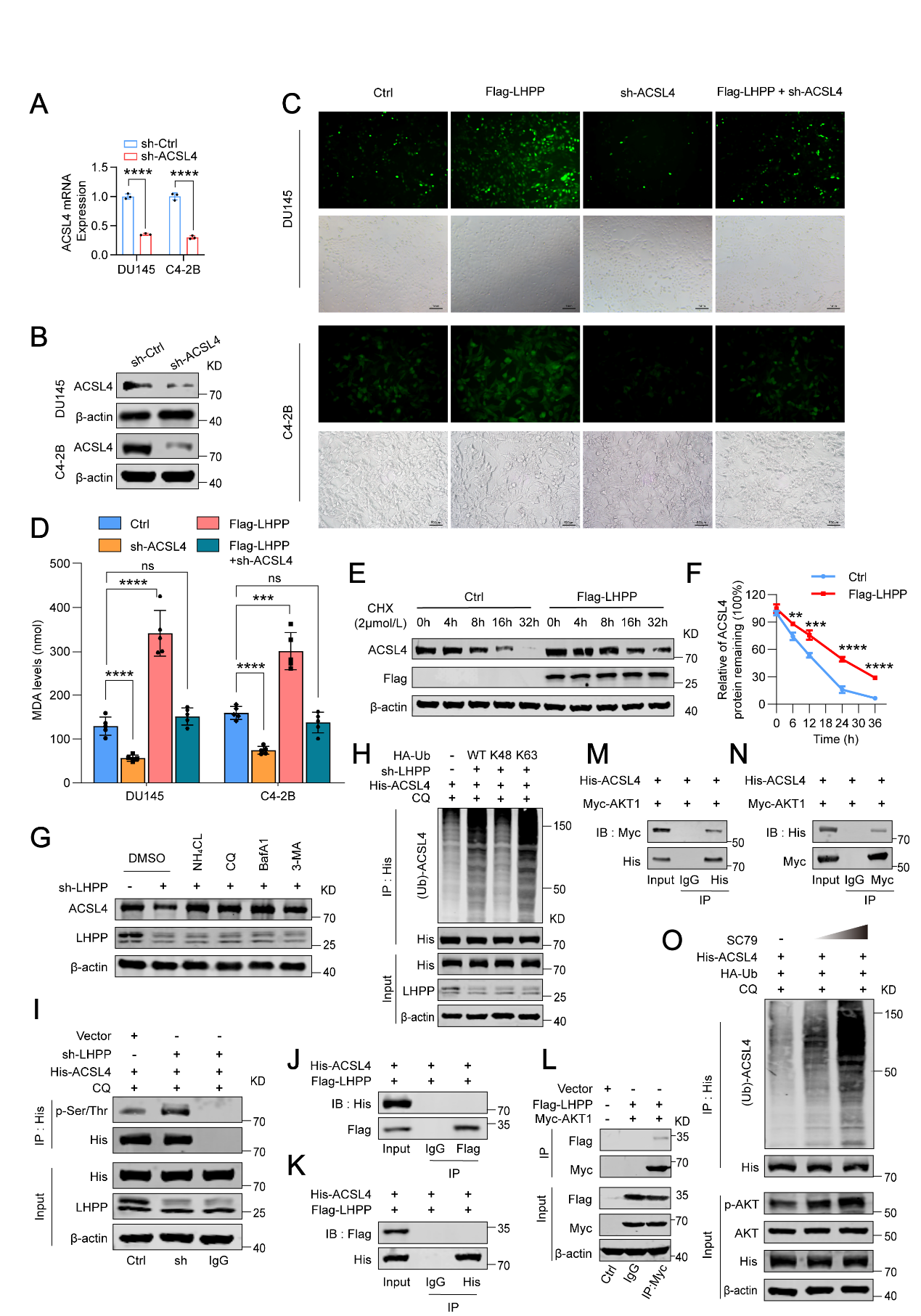


**Supplementary figure 4. LHPP indirectly regulates ACSL4 protein expression via AKT phosphorylation and ubiquitin-lysosome pathway.**

**(A-B)** RT-qPCR and western blot analysis of ACSL4 expression after ACSL4 knockdown in DU145 and C4-2B cell lines. **(C)** Representative images showing ROS in the DU145 and C4-2B cells lines after LHPP overexpression compared to control and ACSL4 knockdown. **(D)** The levels of MDA in DU145 cells lines after overexpression of LHPP compared to control and ACSL4 knockdown. **(E)** Western blot analysis of half-life of ACSL4 protein after LHPP overexpression followed by treatment with CHX (2 μM) for 0, 4, 8, 16, or 36 h. **(F)** The protein bands were quantified and normalized to the band intensity at the 0 h time point. **(G)** Western bolt analysis of the impact of NH_4_CL (10 μM), CQ (10 μM), BafA1 (10 μM) and 3-MA (10 μM) for 12 h on ACSL4 reduction mediated by the knockdown of LHPP. **(H)** Western blot analysis of wild-type, K48, and K63 poly-ubiquitination levels of His-tagged ACSL4 after LHPP knockdown and treatment with CQ (10 μM) for 24 h. **(I)** The phosphorylation levels of His-tagged ACSL4 in DU145 cells was detected by Co-IP following the overexpression of LHPP and treatment with CQ (10 μM) for 24 h. **(J-K)** Western blot analysis of the interaction between Flag-tagged LHPP and His-tagged ACSL4 revealed by Co-IP experiments. (**L)** Western blot analysis of Flag-tagged LHPP’s ability to regulate AKT phosphorylation levels by binding with Myc-tagged AKT1, as identified via co-immunoprecipitation. **(M-N)** Western blot analysis of the interaction between Myc-tagged AKT1 and His-tagged ACSL4 revealed by Co-IP experiments. **(O)** Western blot analysis of K63 poly-ubiquitination levels of His-tagged ACSL4 after SC79 (0, 5,10 μM) and CQ (10 μM) treatment for 24 h. Statistical significance was determined by unpaired t test **(A, D)** or One-way ANOVA **(F)** and data represented as mean ± SD. ns, not significant; ** *p* < 0.01; *** *p* < 0.001; **** *p* < 0.0001, respectively.


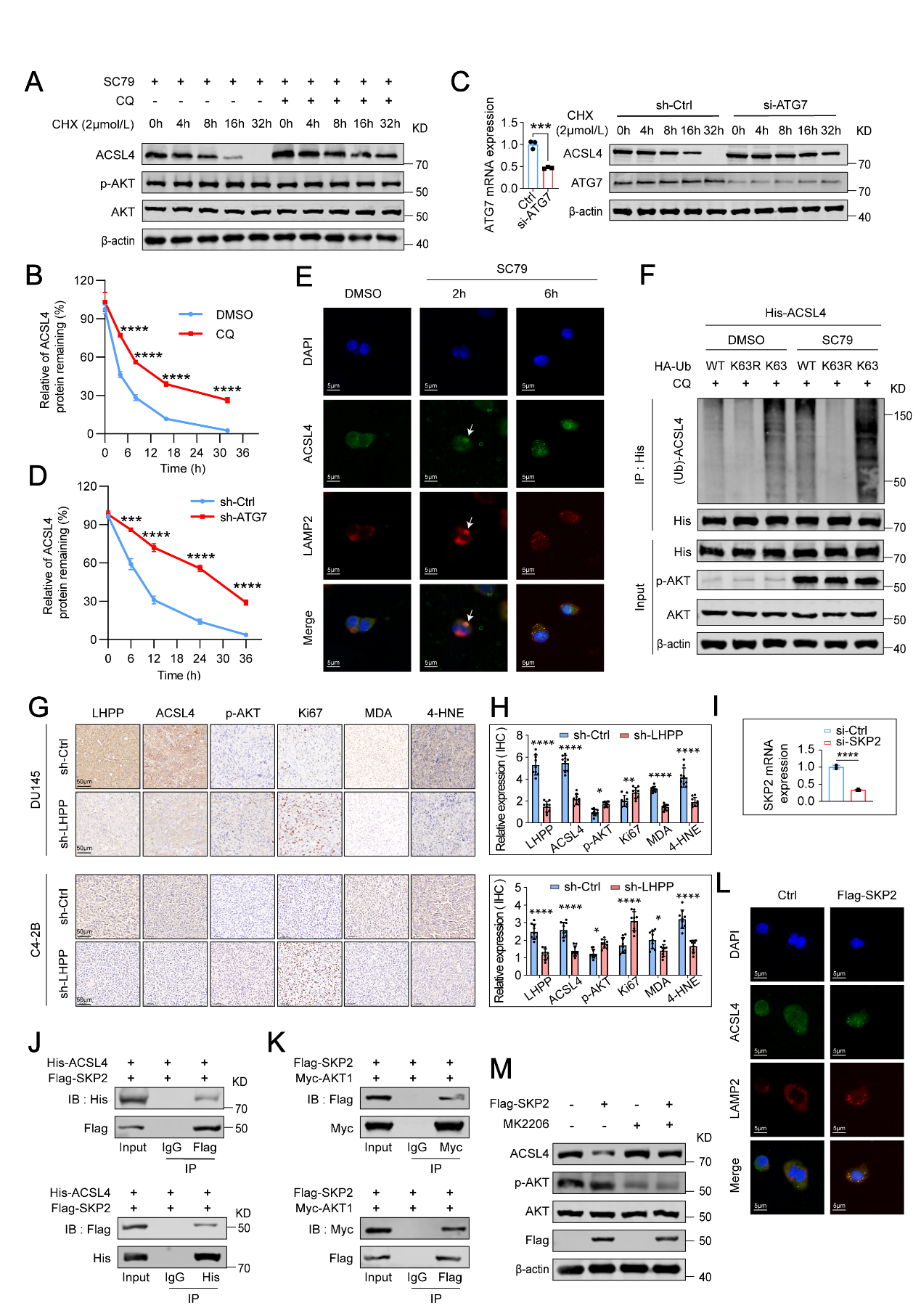


**Supplementary figure 5.** **Role of SKP2 in LHPP/AKT-mediated ACSL4 degradation.**

**(A)** Western blot analysis of the half-life of ACSL4 protein after treatment with SC79 (10 μM) and CQ (10 μM), followed by treatment with CHX (2 μM) for 0, 4, 8, 16, or 36 h. **(B)** The protein bands were quantified and normalized to the band intensity at the 0 h time point. **(C)** RT-qPCR analysis of ATG7 mRNA expression after transfection with siRNA, and western blot analysis of half-life of ACSL4 protein after ATG7 knockdown followed by treatment with CHX (2 μM) for 0, 4, 8, 16, or 36 h. **(D)** The protein bands were quantified and normalized to the band intensity at the 0 h time point. **(E)** Co-localization of ACSL4 and LAMP2 following DMSO or SC79 (10 μM) treatment for 0, 2, or 6 h. **(F)** Western blot analysis of wild-type, K63R, and K63 poly-ubiquitination levels of His-tagged ACSL4 after treatment with SC79 (10 μM) and CQ (10 μM) for 24 h. **(G-H)** Levels of IHC staining of LHPP, ACSL4, p-AKT (S473) Ki67, MDA, and 4-HNE in the tumor model using DU145 and C4-2B cells in control and LHPP knockdown group. **(I)** RT-qPCR analysis of SKP2 mRNA expression after transfection with siRNA. **(J)** Western blot analysis of the interaction between Flag-tagged SKP2 and His-tagged ACSL4 revealed by Co-IP experiments. **(K)** Western blot analysis of the interaction between Flag-tagged SKP2 and Myc-tagged AKT1 revealed by Co-IP experiments. **(L)** Co-localization of ACSL4 and LAMP2 following SKP2 overexpression. **(M)** Western blot analysis of the effects of the AKT inhibitor MK2206 (5 μM) on SKP2-mediated ACSL4 degradation in DU145 cells overexpressing SKP2, with MK2206 significantly inhibiting the degradation process. Statistical significance was determined by unpaired t test **(C, H, I)** or One-way ANOVA **(B, D)** and data are represented as mean ± SD. * *p* < 0.05; ** *p* < 0.01; *** *p* < 0.001; **** *p* < 0.0001, respectively.


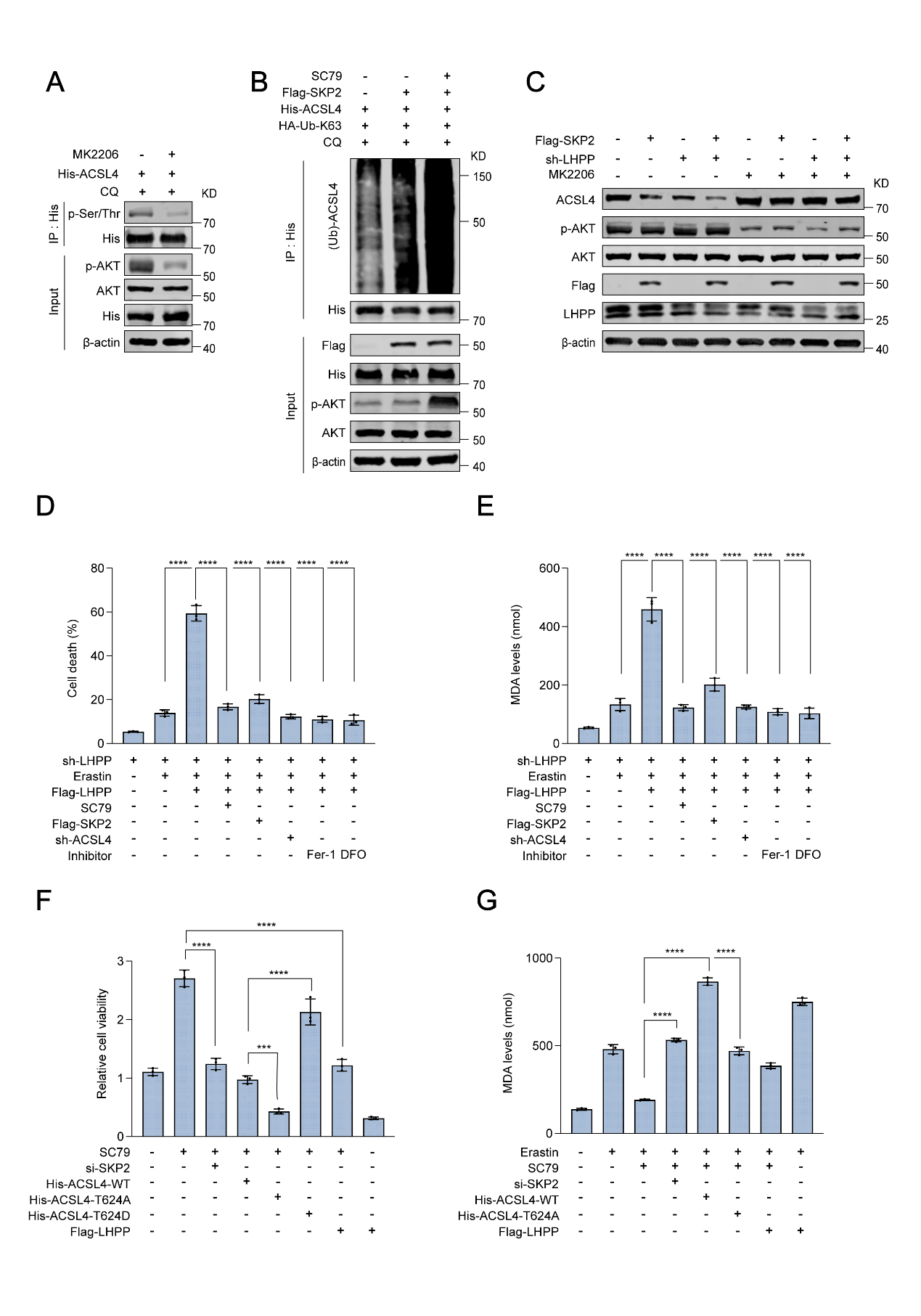


**Supplementary figure 6. LHPP inhibits PCa proliferation by blocking AKT/SKP2-induced ACSL4 degradation to promote ferroptosis.**

**(A)** The phosphorylation status of His-tagged ACSL4 was detected by Co-IP following the treatment with MK2206 (5 μM) for 24 h. **(B)** The augmentation of SKP2-mediated K63-linked poly-ubiquitin degradation of His-tagged ACSL4, following treatment with SC79 (10 μM) and CQ (10 μM) for 24 h, indicates that ACSL4 degradation by SKP2 is contingent upon AKT activation. **(C)** Observation of enhanced SKP2-mediated ACSL4 degradation in LHPP knockdown cells, and the inhibitory effect of MK2206 (5 μM) on this process. **(D)** CCK-8 assay to test cell death in DU145 cells transfected with the indicated vectors. **(E)** MDA assay to test lipid peroxidation in DU145 cells transfected with the indicated vectors. **(F)** CCK-8 assay to test cell viability in DU145 cells transfected with the indicated vectors. **(G)** MDA assay to test lipid peroxidation in DU145 cells transfected with the indicated vectors. Statistical significance was determined by unpaired t test **(D, E, F, G)** and data are represented as mean ± SD. *** *p* < 0.001; **** *p* < 0.0001, respectively.


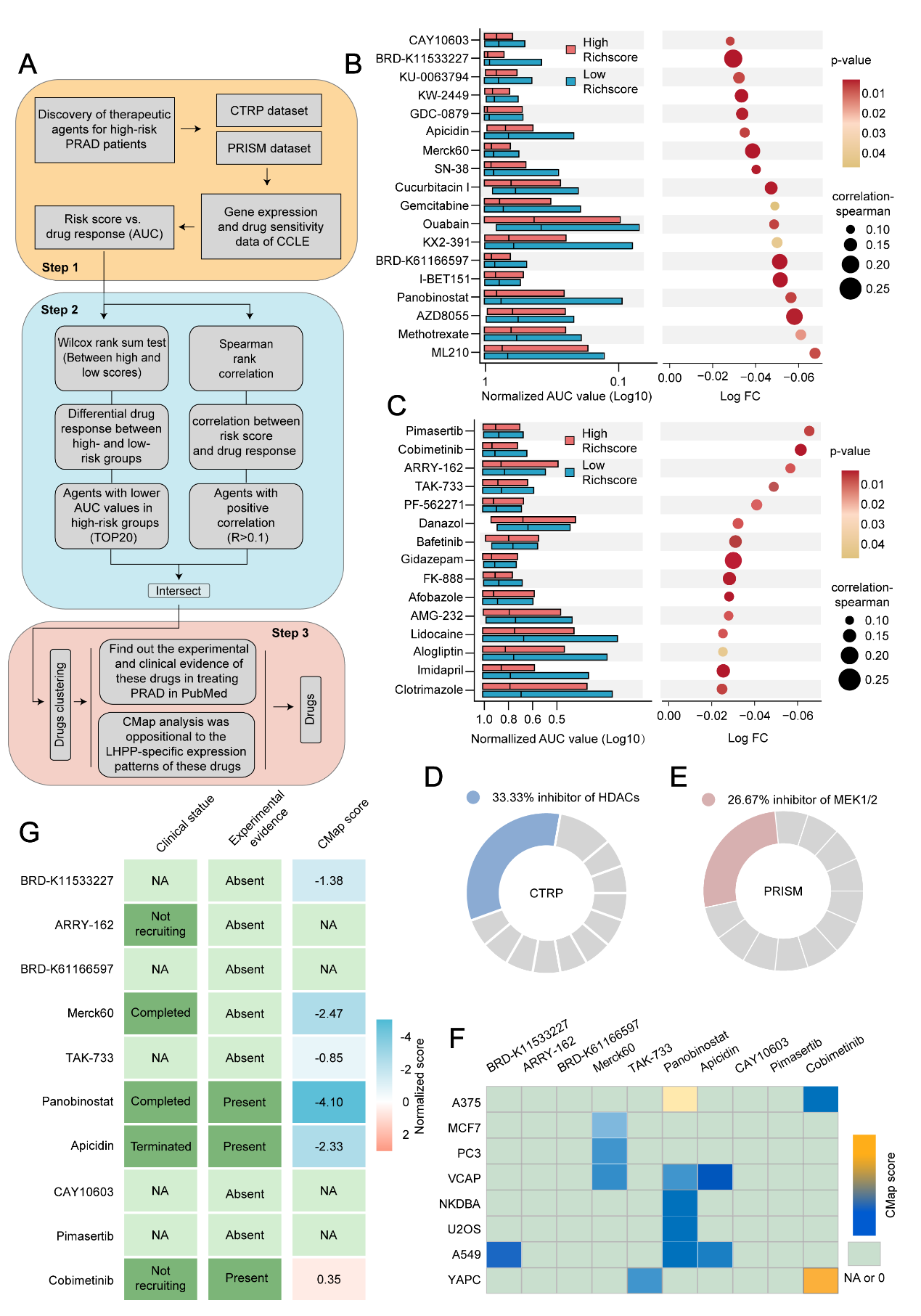


**Supplementary figure 7. Potential therapeutic application of Panobinostat in PCa treatment.**

**(A)** Overview of the analysis procedures. **(B)** Identification of the top 20 compounds with negative Log2 fold change (Log2FC) differentials in CTRPv2 datasets, exhibiting a negative correlation with the LHPP risk score. **(C)** Identification of the top 20 compounds with negative Log2FC differentials in PRISM datasets, exhibiting a negative correlation with the LHPP risk score. **(D-E)** Detailed analysis of selected drugs showing their pharmacological effects, primarily as histone deacetylase inhibitors (HDACi) and mitogenic activated protein kinase inhibitors (MEKi). **(F)** CMap scores for the compounds indicating their potential for treating PCa. **(G)** Identification and evaluation of the most promising subclass-specific therapeutic agents, based on multidimensional evidence.


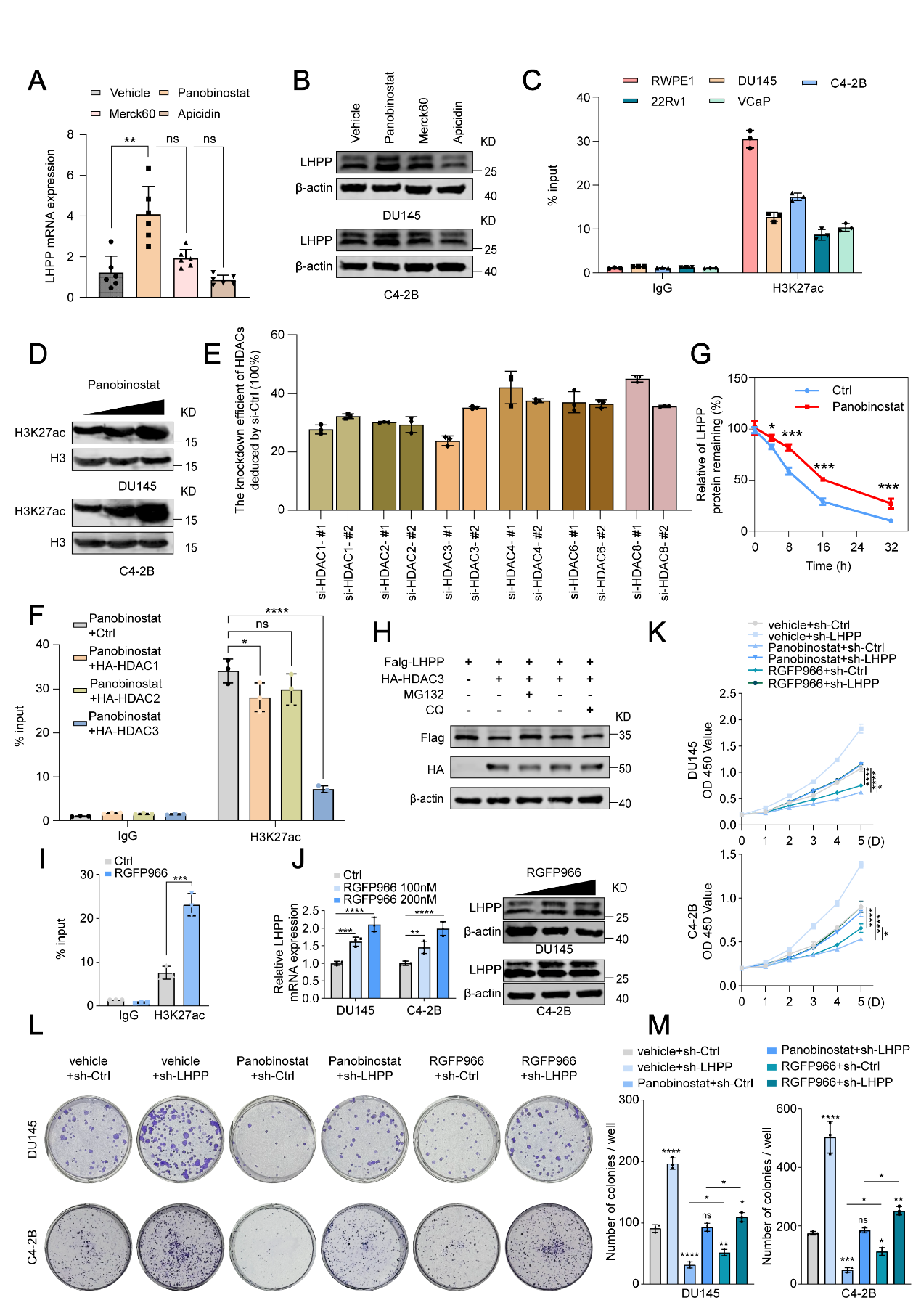


**Supplementary figure 8. Panobinostat enhances LHPP expression through HDAC3 inhibition in PCa.**

**(A-B)** The impact on LHPP expression through RT-qPCR and western blot analysis, following the compounds DMSO, Panobinostat, Merck60, and Apicidin treatment for 48 h. **(C)** ChIP-qPCR analysis of H3K27ac enrichment at the LHPP promoter in normal prostate cell lines RWPE-1 and PCa cell lines DU145, C4-2B, 22Rv1, and VCaP. **(D)** Western blot analysis of the levels of H3K27ac after the treatment with Panobinostat (0, 50, 100 nM) for 48 h in DU145 and C4-2B cells. **(E)** The knockdown efficiency of each member of class I/II HDACs by siRNAs. Expression of each gene was first normalized to the level of β-actin. **(F)** ChIP-qPCR analysis of H3K27ac enrichment at the LHPP promoter with the following Panobinostat treatment in DU145 cells with overexpressed HDACs. **(G)** The protein bands were quantified and normalized to the band intensity at the 0 h time point. **(H)** Western blot analysis of the level of Flag-tagged LHPP after HDAC3 overexpression, and treatment with MG132 (4 μM) and CQ (10 μM) for 24 h. **(I)** ChIP-qPCR analysis of H3K27ac enrichment at the LHPP promoter after RGFP966 (200 nM) treatment for 24 h in DU145 cells. **(J)** Significant increase in LHPP mRNA and protein expression in DU145 and C4-2B cell lines after RGFP966 (200 nM) treatment for 48 h. **(K)** The effect of Panobinostat (100 nM) or RGFP966 (200 nM) treatment on cell viability in DU145 and C4-2B cells was modulated with or without LHPP knockdown. **(L-M)** The colony formation assays of the effect of Panobinostat (100 nM) or RGFP966 (200 nM) treatment in DU145 and C4-2B cells were modulated with or without LHPP knockdown. Statistical significance was determined by unpaired t test **(A, F, I, J, M)** or One-way ANOVA **(G, K, J)** and data are represented as mean ± SD. ns, not significant; * *p* < 0.05; ** *p* < 0.01; *** *p* < 0.001; **** *p* < 0.0001, respectively.


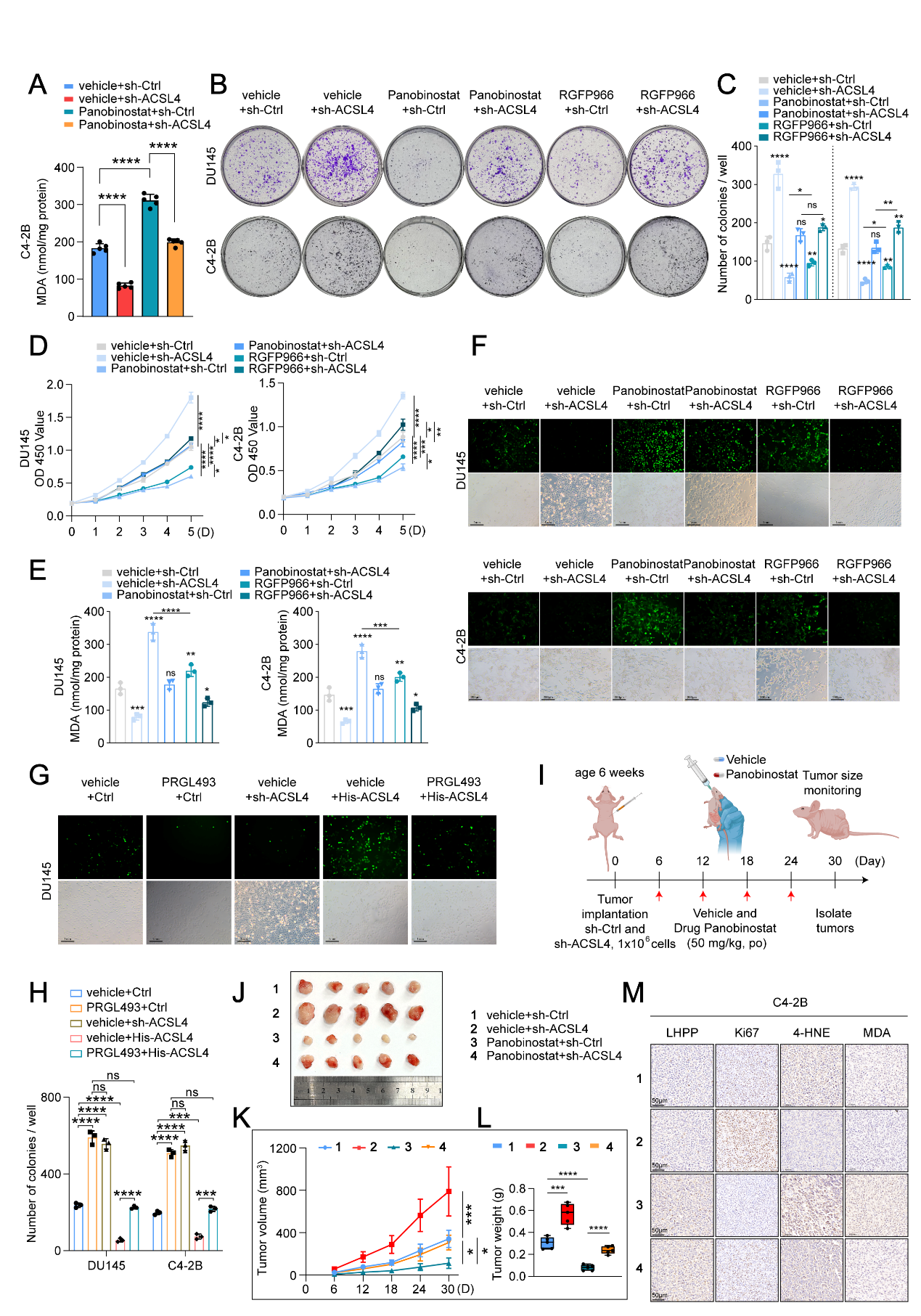


**Supplementary figure 9. Panobinostat modulates LHPP and ACSL4-driven ferroptosis to arrest the advancement of prostate cancer.**

**(A)** The levels of MDA in C4-2B cells treated with Panobinostat (100 nM) for 24 h compared to control and ACSL4 knockdown cells. **(B-C)** The colony formation assays of the effect of Panobinostat (100 nM) or RGFP966 (200 nM) treatment in DU145 and C4-2B cells were modulated with or without ACSL4 knockdown. **(D)** The effect of Panobinostat (100 nM) or RGFP966 (200 nM) treatment on cell viability in DU145 and C4-2B cells were modulated with or without ACSL4 knockdown. **(E)** The levels of MDA in DU145 and C4-2B cells treated with Panobinostat (100 nM) or RGFP966 (200 nM) for 24 h compared to control and ACSL4 knockdown cells. **(F)** Representative images showing ROS in the DU145 and C4-2B cells lines after treatment with Panobinostat (100 nM) or RGFP966 (200 nM) for 24 h compared to control and ACSL4 knockdown. **(G)** The colony formation assays of the effect of PRGL493 (5 μM) treatment in DU145 and C4-2B cells was modulated with or without ACSL4 knockdown and overexpression. **(H)** Representative images showing ROS in the DU145 cells lines after treatment with PRGL493 (5 μM) for 24 h compared to control and ACSL4 knockdown and overexpression. **I-L** Representative images illustrate *in vivo* experiments using a nude mouse model with subcutaneous tumors derived from the control C4-2B cell line and ACSL4 knockdown C4-2B cells, demonstrating the inhibitory effect of orally administered Panobinostat on tumor growth, and reversal of this effect with ACSL4 knockdown. The tumor growth (**J**), tumor volume (**K**), and tumor weight (**L**) were monitored. **(M)** Levels of IHC staining of LHPP, Ki67, MDA, and 4-HNE in the tumor model using C4-2B cells. Statistical significance was determined by unpaired t test **(A, C, E, H, L)** or One-way ANOVA **(D, K)** and data are represented as mean ± SD. ns, not significant; * *p* < 0.05; ** *p* < 0.01; *** *p* < 0.001; **** *p* < 0.0001, respectively.

**Supplementary Table**

**Table S1. Clinical characteristics of PARD patients**

| **Characteristics** | **Number of patients (%)** |
| --- | --- |
| **Gender** |  |
| Male | 70 (100%) |
| **Age** |  |
| ≥60 | 52 (74.29%) |
| <60 | 18 (25.71%) |
| **Clinical T state** |  |
| 1 | 1 (1.43%) |
| 2 | 26 (37.14%) |
| 3 | 33 (47.14%) |
| 4 | 10 (14.29%) |
| **Clinical N state** |  |
| 0 | 42 (60.00%) |
| 1 | 28 (40.00%) |
| **Clinical M state** |  |
| 0 | 61 (87.14%) |
| 1 | 9 (12.86%) |
| **Gleason score** |  |
| 6 | 24 (34.29%) |
| 7 | 26 (37.14%) |
| 8 | 12 (17.14%) |
| 9 | 8 (11.43%) |

**Table S2. Primer shRNA and siRNA sequence information**

| **Use** | **Product name** | **Forward primers (5’-3’)** |
| --- | --- | --- |
| Human siRNA | si-HDAC1 #1 | CGGUUAGGUUGCUUCAAUCUA |
|  | si-HDAC1 #2 | CGGUCAUGUCCAAAGUAAUTT |
|  | si-HDAC2 #1 | AAGCCUCAUAGAAUCCGCAUG |
|  | si-HDAC2 #2 | UGUGAAGUUAAACCGACAATT |
|  | si-HDAC3 #1 | GGAAAGCGAUGUGGAGAUU |
|  | si-HDAC3 #2 | AAAGCGAUGUGGAGAUUUA |
|  | si-HDAC4 #1 | UGUGAAGUUAAACCGACAATT |
|  | si-HDAC4 #2 | GGAAUCUGAACCACUGCAUTT |
|  | si-HDAC6 #1 | GCAAUGGAAGAAGACCUAATT |
|  | si-HDAC6 #2 | GGATGGATCTGAACCTTGAGA |
|  | si-HDAC8 #1 | GGUCCCGGUUUAUAUCUAUTT |
|  | si-HDAC8 #2 | GGUGUACAUAGCCUUUAAUTT |
|  | si-SKP2 | GGAUGUGACUGGUCGGUUG |
|  | si-ACSL4 | GGGAGUGAUGAUGCAUCAUAGCAAU |
|  | si-HMOX1 | GACUGCGUUCCUGCUCAAC |
|  | si-ATG7-F | GCUCUUCCUUACUUCUUAA |
|  | si-ATG7-R | UUAAGAAGUAAGGAAGAGC |
|  | si-Ctrl | UUCUCCGAACGUGUCACGU |
| Human shRNA | sh-LHPP #1 | CAACCCAAACTGTGTGGTAAT |
|  | sh-LHPP #2 | AGTATGCCTGTGGCATCAAAG |
|  | sh-LHPP #3 | ACCACTCACCATGGGCCTTTA |
|  | sh-ACSL4 | GCAGTAGTTCATGGGCTAAAT |
|  | sh-Ctrl | CCTAAGGTTAAGTCGCCCTCG |
| Human RNA qPCR | LHPP -Forward | CACCAACGAGTCGCAGAAGTCC |
|  | LHPP -Reverse | CCTCCTGCTCAGAGATGTCAAATCC |
|  | AKT1 -Forward | CCACGCTACTTCCTCCTCAAGAATG |
|  | AKT1 -Reverse | CCATCTCCTCCTCCTCCTGCTTC |
|  | ACSL4 -Forward | CATCCCTGGAGCAGATACTCT |
|  | ACSL4 -Reverse | TCACTTAGGATTTCCCTGGTCC |
|  | GAPDH -Forward | TGACATCAAGAAGGTGGTGAAGCAG |
|  | GAPDH -Reverse | GTGTCGCTGTTGAAGTCAGAGGAG |
|  | β-actin -Forward | CAGATGTGGATCAGCAAGCAGGAG |
|  | β-actin -Reverse | CGCAACTAAGTCATAGTCCGCCTAG |
|  | CD44 -Forward | GGGAGTCAAGAAGGTGGAGCAAAC |
|  | CD44 -Reverse | GCCAAGAGGGATGCCAAGATGATC |
|  | HMOX1 -Forward | TGCCAGTGCCACCAAGTTCAAG |
|  | HMOX1 -Reverse | TGTTGAGCAGGAACGCAGTCTTG |
|  | ATG7 -Forward | GCCGTGGAATTG ATGGTATC |
|  | ATG7 -Reverse | GCCGTGGAATTGATGGTATC |
|  | NDRG1 -Forward | CTTGTGCGGAAGGCTGGATGG |
|  | NDRG1 -Reverse | TGCTGGCGGTAGGTGTGGAC |
|  | HDAC1 -Forward | CGCCCTCACAAAGCCAATG |
|  | HDAC1 -Reverse | CTGCTTGCTGTACTCCGACA |
|  | HDAC2 -Forward | ATGGCGTACAGTCAAGGAGG |
|  | HDAC2 -Reverse | TGCGGATTCTATGAGGCTTCA |
|  | HDAC3 -Forward | TCTGGCTTCTGCTATGTCAACG |
|  | HDAC3 -Reverse | CCCGGTCAGTGAGGTAGAAAG |
|  | HDAC4 -Forward | GCCAAAGATGACTTCCCTCTTA |
|  | HDAC4 -Reverse | TTTCGGCCACTTTCTGCTTTAG |
|  | HDAC6 -Forward | AAGAAGACCTAATCGTGGGACT |
|  | HDAC6 -Reverse | GCTGTGAACCAACATCAGCTC |
|  | HDAC8 -Forward | CATCATGCAAAGAAAGATGA |
|  | HDAC8 -Reverse | CTGAAGGCATCTTCTACACC |
| ChIP-qPCR | H3K27ac -Forward | GTGCTGCTTGACATCTCGGG |
|  | H3K27ac -Reverse | CTGAGCTTAGAGCTCGGCGG |

**Supplementary materials and methods**

**Label-Free Mass spectrometry**

Approximately 10 million DU145-sh-Ctrl and DU145-sh-LHPP stable cell samples were harvested and lysed using RIPA buffer containing protease and phosphatase inhibitors. The lysates were centrifuged at 14,000 × g for 20 minutes, and the supernatants were collected. These were treated with 200 mM dithiothreitol (DTT) at 37°C for 1 hour, diluted four-fold with 25 mM ammonium bicarbonate (ABC), and digested overnight with trypsin at a 1:50 trypsin-to-protein ratio at 37°C. Digestion was halted by adding 50 μl of 0.1% formic acid (FA).

For solid-phase extraction, the C18 columns were prepped by washing with 100 μl of 100% acetonitrile (ACN) and equilibrated with 0.1% FA, followed by centrifugation at 1200 rpm for 3 minutes for each step. Samples were then loaded onto the columns, washed twice with 0.1% FA, and then once with pH 10 water, with centrifugation after each wash. Peptides were eluted with 70% ACN, collected, lyophilized, and stored at -80°C until further analysis.

Nanoflow LC-MS/MS analysis of the tryptic peptides was performed on a Thermo Scientific Orbitrap Exploris™ 480 mass spectrometer connected to an EASY nLC 1200 system. We loaded 500 ng of peptides onto a 25 cm column with 1.9-μm ReproSil-Pur C18-AQ silica beads. The peptides were separated over a 120-minute gradient at 600 nl/min, starting with 8% buffer B (80% ACN, 0.1% FA), increasing to 30% over 79 minutes, then to 40% over 16 minutes, ramping to 95% in 1 minute, and holding at 95% for 4 minutes.

MS2 spectra were matched to the UniProtKB human proteome database (20,373 sequences, retrieved March 17, 2022) using the Sequest HT search engine. Parameters included full tryptic specificity, up to two missed cleavages, a minimum peptide length of 6, fixed carbamidomethylation of cysteine (+57.02146 Da), variable oxidation of methionine (+15.99492 Da), a precursor mass tolerance of 15 ppm, and a fragment mass tolerance of 0.02 Da. Protein assembly was based on peptide probabilities, with a final false discovery rate set at 1%.

**Colony formation assay**

Cells were seeded in 6-well plates and cultured over a 14-day timeframe. Following fixation by paraformaldehyde for 30 minutes, cell colonies were stained using crystal violet for a duration of 2 hours. Images of the colonies were obtained through a Leica DM IRB microscope (Wetzlar, Germany). The resultant quantitative data was processed using ImageJ software.

**Cytotoxicity assay**

Cells were introduced into 96-well plates and left to fully adhere before the medium was replaced and cells were exposed to the relevant compounds for a 24-hour period. For the CCK-8 assay, the cell media in each well was substituted with 100 μl of fresh media and 10 μl of CCK-8 reagent (Dojindo, Tokyo, Japan), followed by a one-hour incubation. Optical density at 450 nm (OD450) was then determined using a DNM-9606 microplate reader (Perlong, Beijing, China).

**Scratch assay**

Cells were seeded into 6-well plates and incubated for 24 hours. Subsequently, a sterile 200 μl pipette tip was employed to generate a uniform scratch in the monolayer. Wound closure was monitored at predetermined time points by capturing images with an inverted phase-contrast microscope (Leica DM IRB). The area of the cell-free wounds was quantified using ImageJ software.

**Transwell assay**

Cells were seeded into the upper compartment of a Matrigel-precoated (Becton, Dickinson and Company, USA) transwell apparatus (8.0 μm pore size, 353097, Falcon, Corning, NY) containing 200 μl of serum-free medium. The lower compartment was supplemented with 500 μl of medium enriched with 20% fetal bovine serum (FBS). Following the incubation period, non-migrated cells were gently removed from the upper chamber. Cells that had migrated through the membrane were fixed and stained with 0.1% crystal violet. Migration was documented using a Leica DM IRB phase-contrast microscope, and the number of migrated cells was quantified with ImageJ software.

**MDA assay**

A Solarbio Micro-MDA Assay Kit (BC0025) was utilized to assess lipid peroxidation within cell membranes. After centrifugation to eliminate the supernatant, cells were resuspended, and for every 5 million cells, 1 ml of Reagent I was introduced. Ultrasonication disrupted the cells (20% power, 3-second sonication, 10-second rest, 30 cycles). The resultant suspension was centrifuged at 8000 × g for 10 minutes at 4°C, and the clear supernatant was kept on ice. The spectrophotometer, pre-warmed to 415 nm, facilitated absorbance readings at 450, 532, and 600 nm for 200 μl aliquots from each sample, placed in a 96-well plate. This process enabled the determination of the MDA concentration.

**5-ethynyl-2’-deoxyuridine (EdU) proliferation assay**

The proliferation rate of prostate cancer cells was assessed by employing an EdU Cell Proliferation Image Kit (Abbkine, KTA2031). Cells were distributed at a density of 2 × 10^4^ cells per well across 96-well plates and incubated for a period of 24 hours. Subsequently, an EDU working solution was added for labelling purposes, followed by a further two-hour incubation. Cells were then fixed and permeabilised via a formaldehyde and triton reagent. After a 30-minute exposure to 100 μl of the Click-iT mixture, cells were stained with DAPI for the purpose of nuclear visualisation. Lastly, images were captured and cellular counts obtained using an electron fluorescence microscope.
