# Supplementary figures and images for: Phosphatase LHPP confers prostate cancer ferroptosis activation by modulating the AKT-SKP2-ACSL4 pathway
Source: Cell Death Dis. 2024 Sep 11;15(9):665. doi: 10.1038/s41419-024-07007-8 (PMC11390745; doi:10.1038/s41419-024-07007-8)

Full unedited gel for Figure 1

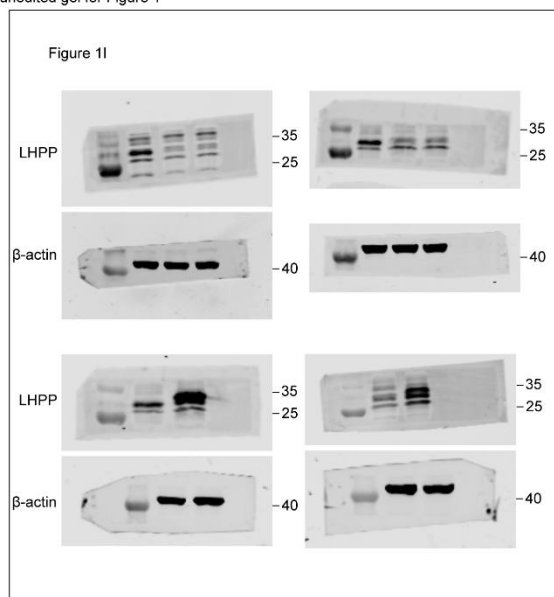

Full unedited gel for Figure 2

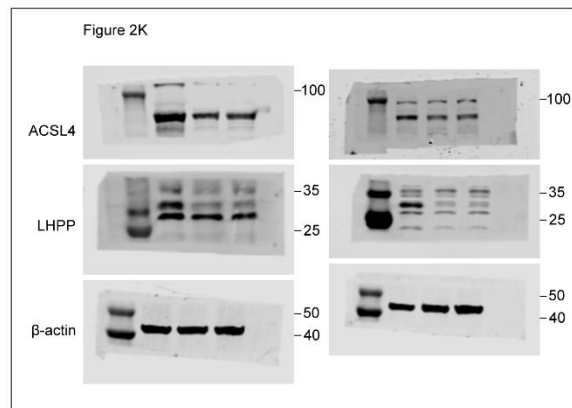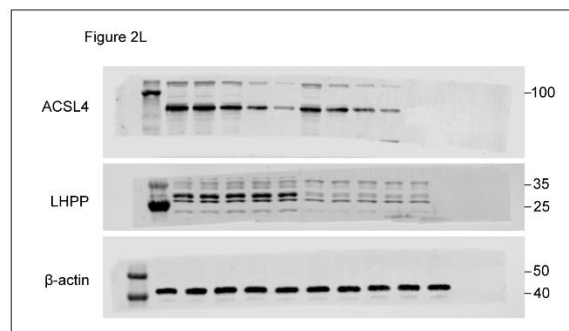

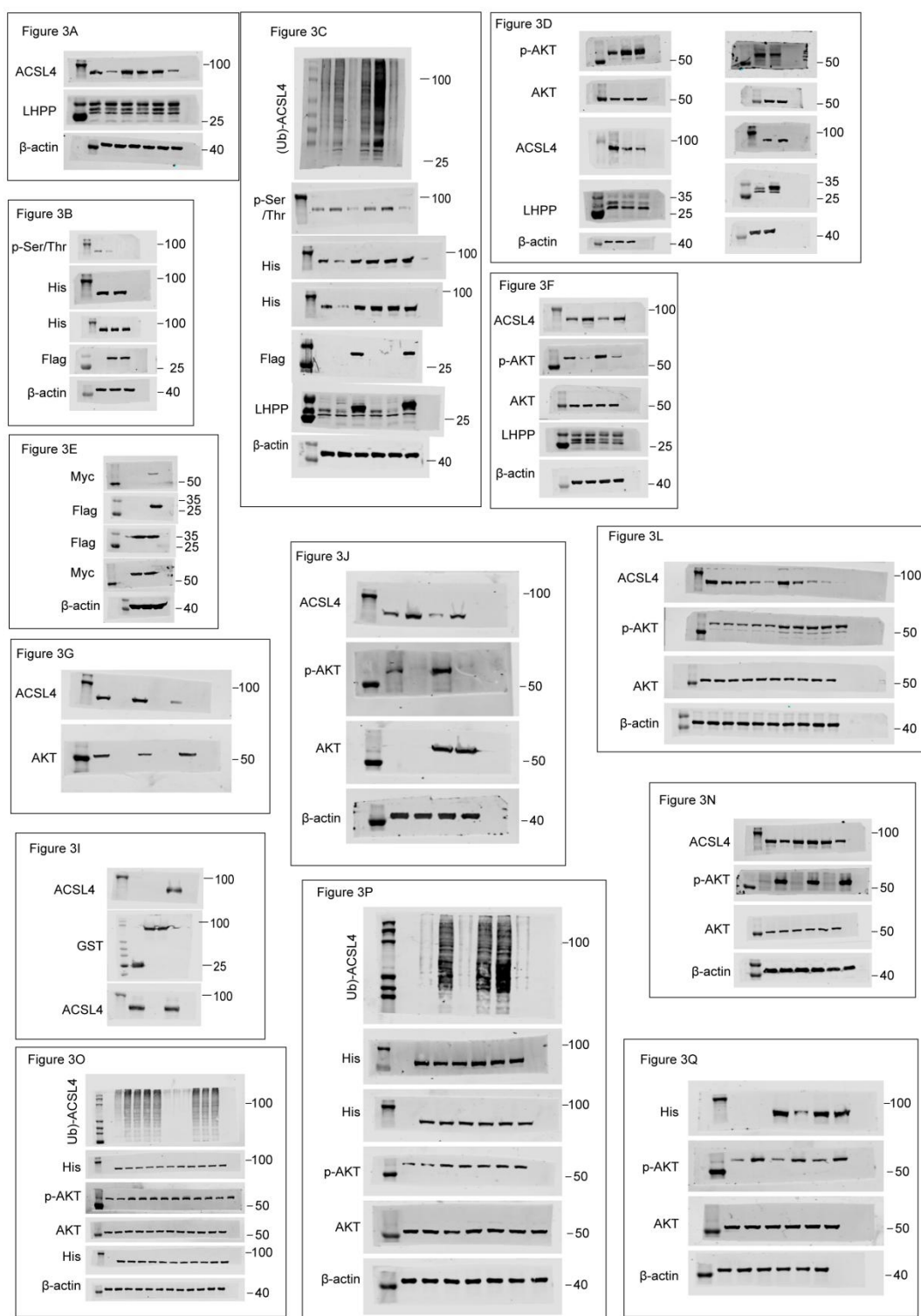

Full unedited gel for Figure 4

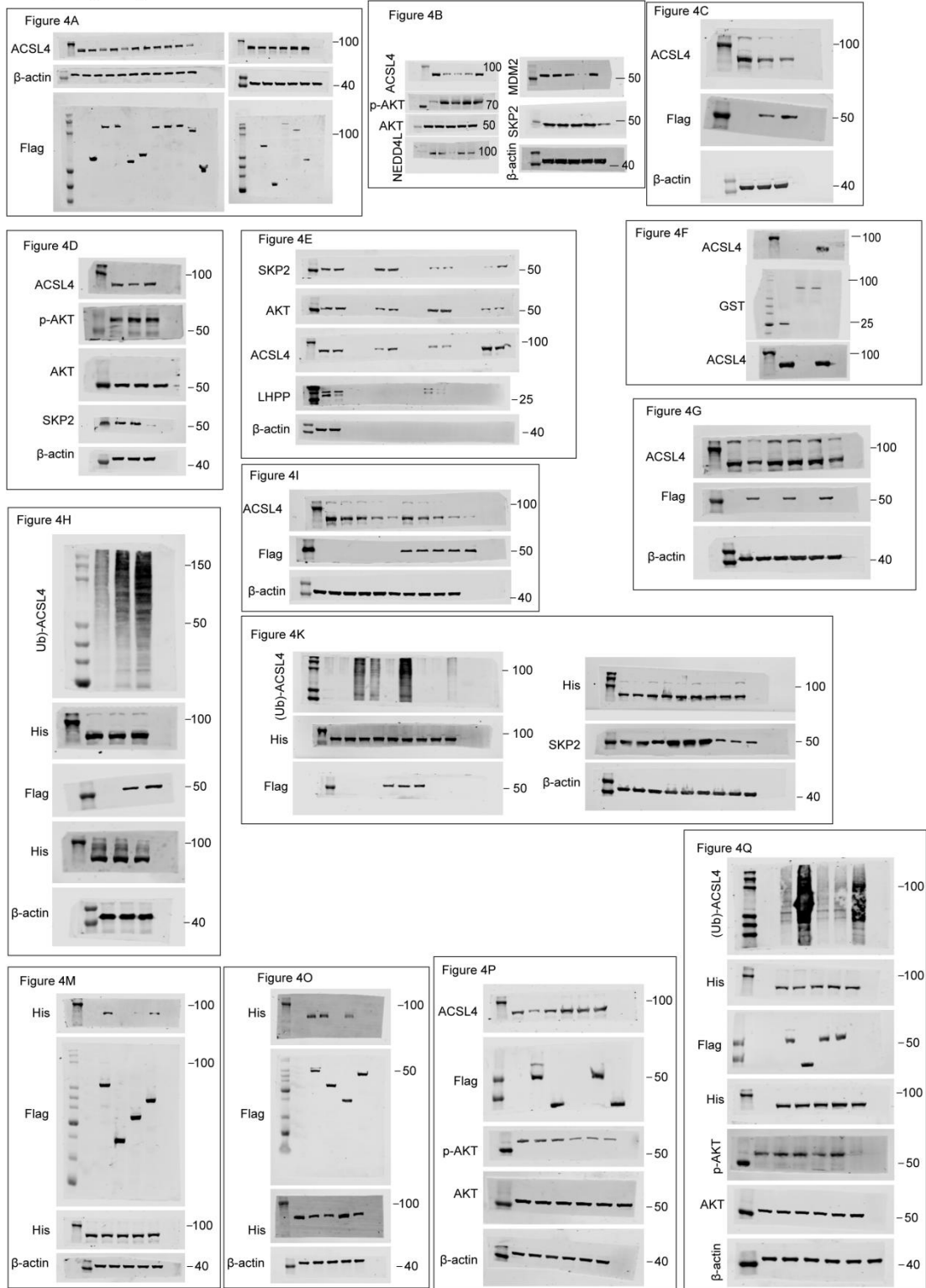

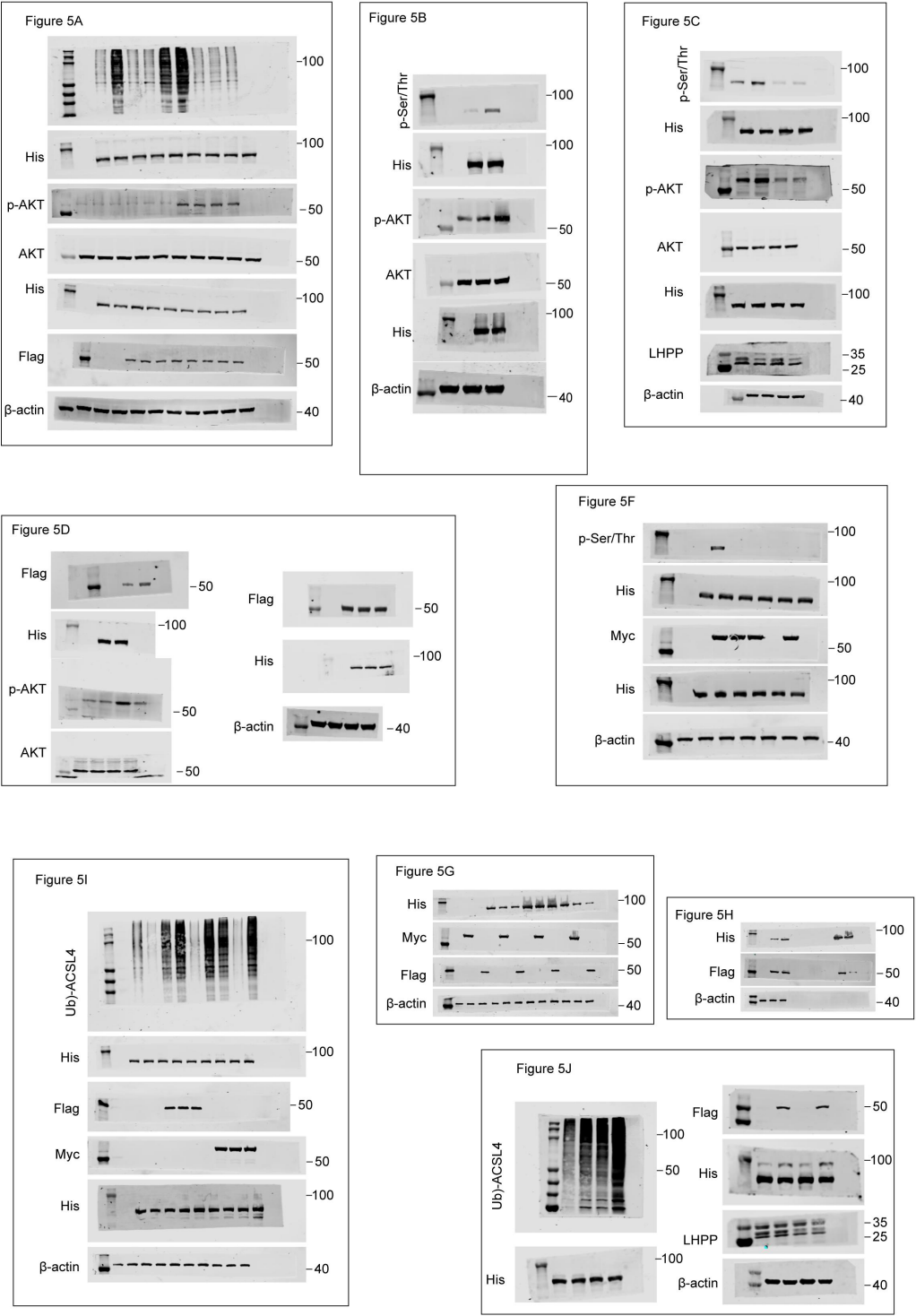

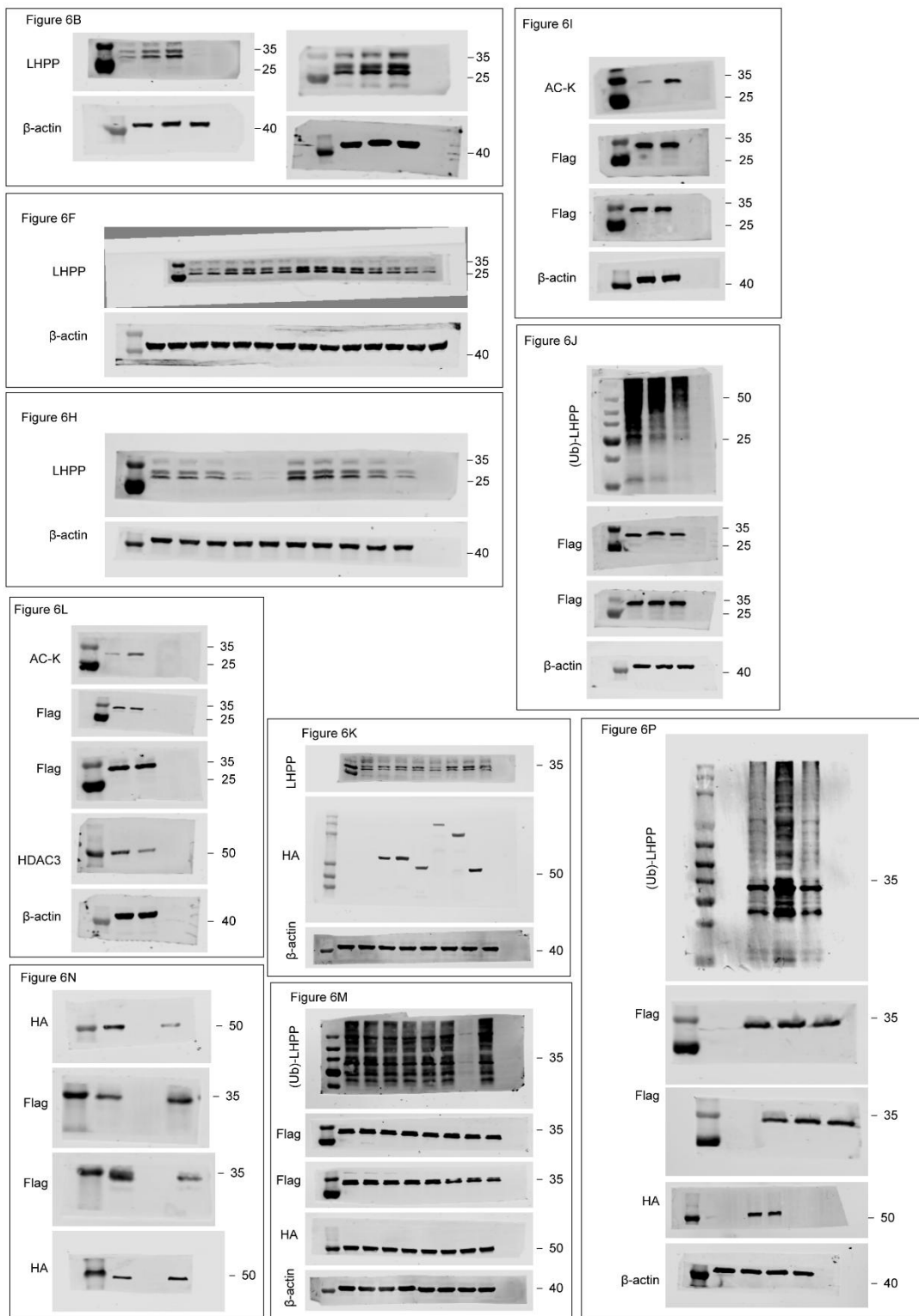

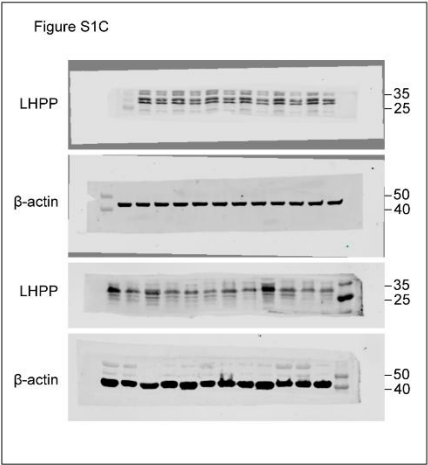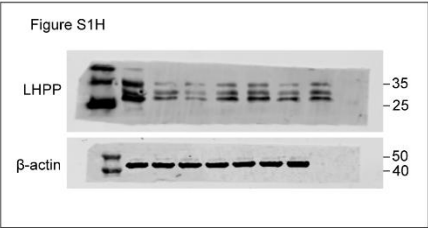

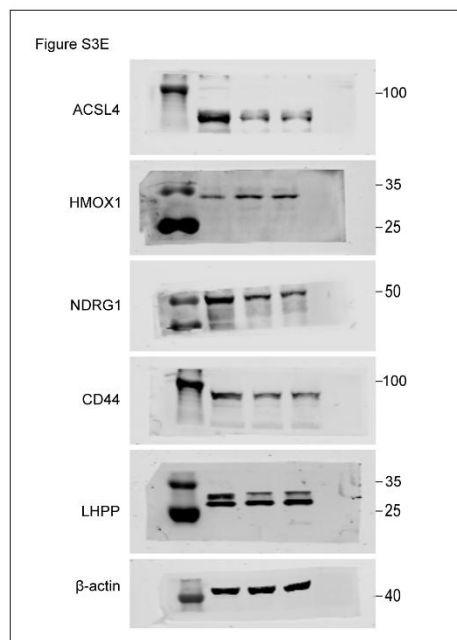

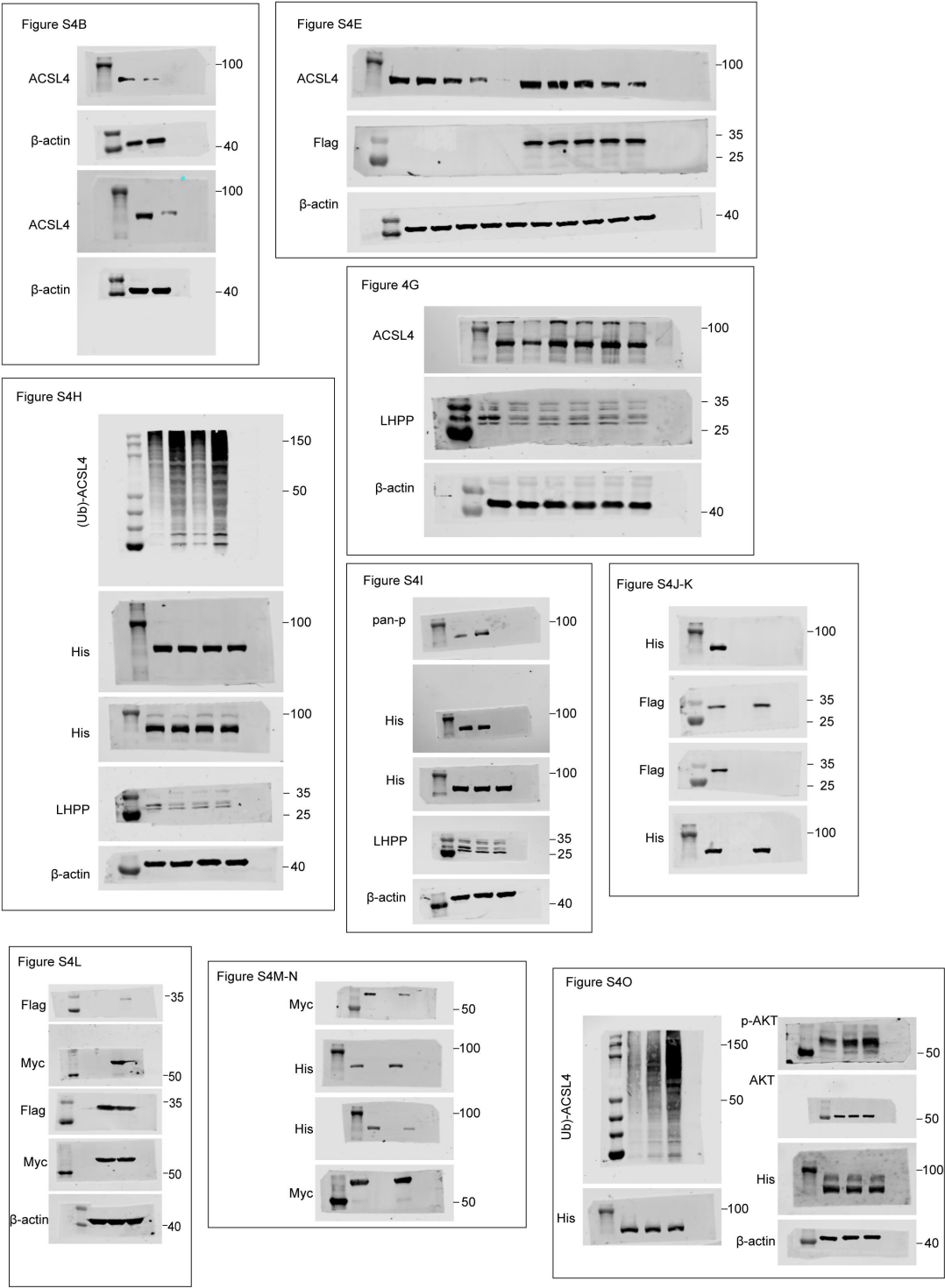

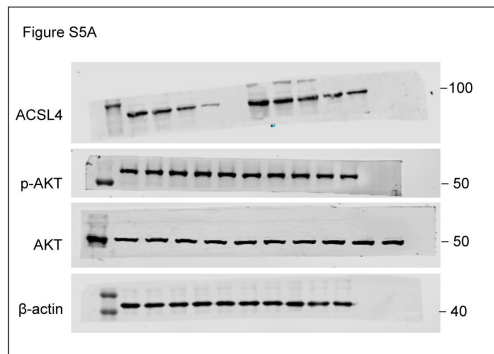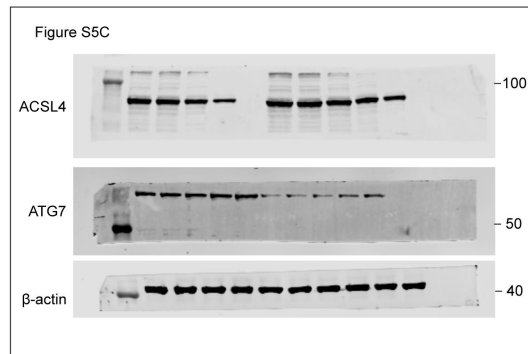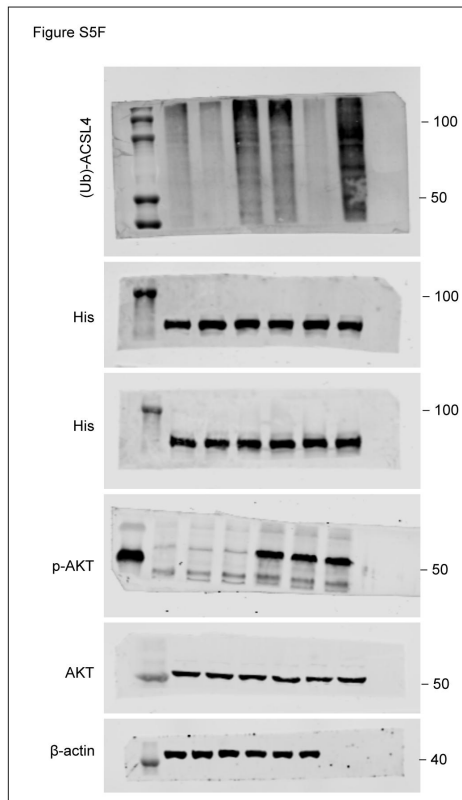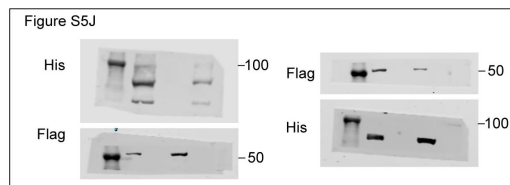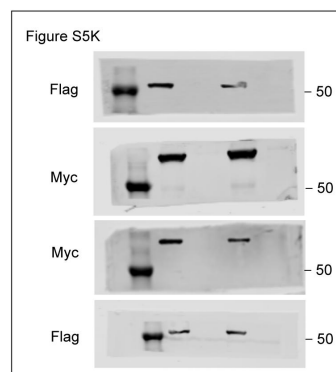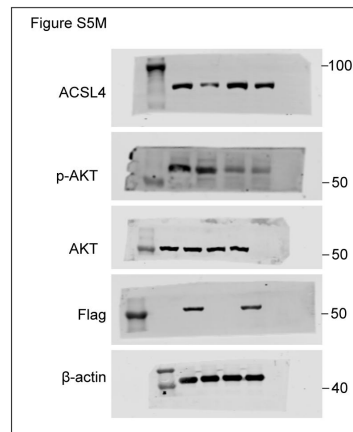

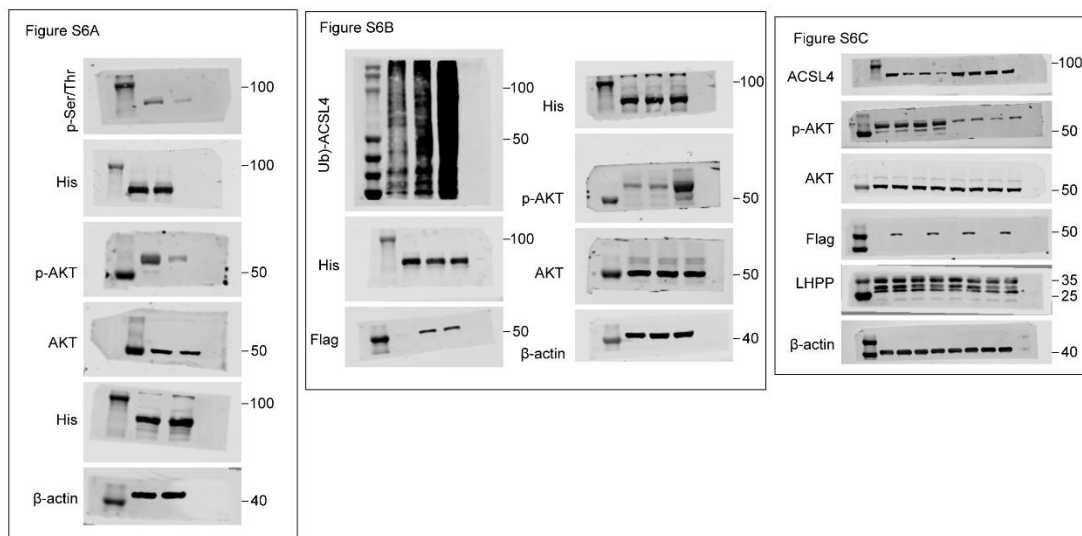

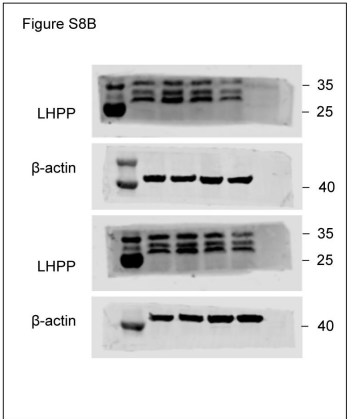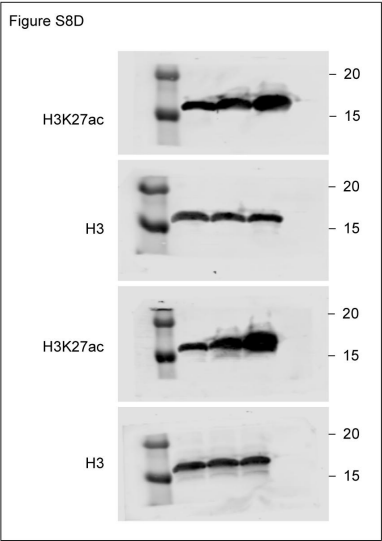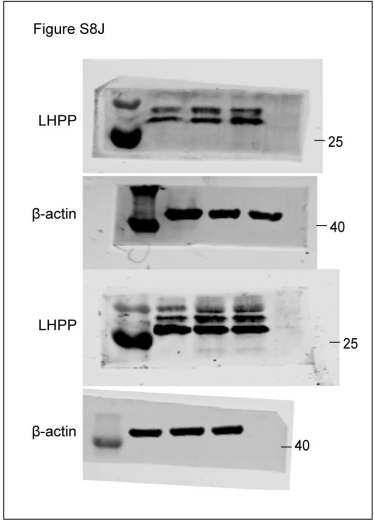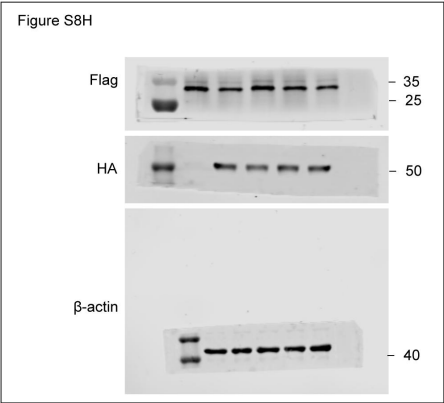

Supplement: Supplementary file 2 — Unedited blot and gel images [file 41419_2024_7007_MOESM2_ESM.pdf]
